# Comparative Genomic and Functional Analysis of 100 *Lactobacillus rhamnosus* Strains and Their Comparison with Strain GG

**DOI:** 10.1371/journal.pgen.1003683

**Published:** 2013-08-15

**Authors:** François P. Douillard, Angela Ribbera, Ravi Kant, Taija E. Pietilä, Hanna M. Järvinen, Marcel Messing, Cinzia L. Randazzo, Lars Paulin, Pia Laine, Jarmo Ritari, Cinzia Caggia, Tanja Lähteinen, Stan J. J. Brouns, Reetta Satokari, Ingemar von Ossowski, Justus Reunanen, Airi Palva, Willem M. de Vos

**Affiliations:** 1 Department of Veterinary Biosciences, University of Helsinki, Helsinki, Finland; 2 Laboratory of Microbiology, Wageningen University, Wageningen, The Netherlands; 3 Infection Biology Program, Department of Bacteriology and Immunology, Haartman Institute, University of Helsinki, Helsinki, Finland; 4 Department of Agri-Food and Environmental System Management, University of Catania, Catania, Italy; 5 Institute of Biotechnology, University of Helsinki, Helsinki, Finland; 6 Functional Foods Forum, University of Turku, Turku, Finland; MicroTrek Incorporated, United States of America

## Abstract

*Lactobacillus rhamnosus* is a lactic acid bacterium that is found in a large variety of ecological habitats, including artisanal and industrial dairy products, the oral cavity, intestinal tract or vagina. To gain insights into the genetic complexity and ecological versatility of the species *L. rhamnosus*, we examined the genomes and phenotypes of 100 *L. rhamnosus* strains isolated from diverse sources. The genomes of 100 *L. rhamnosus* strains were mapped onto the *L. rhamnosus* GG reference genome. These strains were phenotypically characterized for a wide range of metabolic, antagonistic, signalling and functional properties. Phylogenomic analysis showed multiple groupings of the species that could partly be associated with their ecological niches. We identified 17 highly variable regions that encode functions related to lifestyle, *i.e.* carbohydrate transport and metabolism, production of mucus-binding pili, bile salt resistance, prophages and CRISPR adaptive immunity. Integration of the phenotypic and genomic data revealed that some *L. rhamnosus* strains possibly resided in multiple niches, illustrating the dynamics of bacterial habitats. The present study showed two distinctive geno-phenotypes in the *L. rhamnosus* species. The geno-phenotype A suggests an adaptation to stable nutrient-rich niches, *i.e.* milk-derivative products, reflected by the alteration or loss of biological functions associated with antimicrobial activity spectrum, stress resistance, adaptability and fitness to a distinctive range of habitats. In contrast, the geno-phenotype B displays adequate traits to a variable environment, such as the intestinal tract, in terms of nutrient resources, bacterial population density and host effects.

## Author Summary

Some bacterial species are specialists and adapted to a single niche, while others are generalists and able to grow in various environmental conditions. *Lactobacillus rhamnosus* is a generalist and its members can often be found in different human cavities but also in various artisanal and industrial dairy products. To gain insights into the genetic complexity and ecological versatility of this species, we collected 100 *L. rhamnosus* strains from different niches. Genomic and functional analysis of these revealed a dichotomy within the species that reflected its adaptation to particular niches. The variable regions identified in the *L. rhamnosus* genome encode lifestyle traits that allowed us to demonstrate that some *L. rhamnosus* isolates possibly resided in multiple habitats. Our work brings valuable data on the ecological dynamics and adaptability of the species and provides a basis for a model explaining the ecology of *L. rhamnosus* in an anthropocentric perspective. Finally, we observed that a set of pheno-genomic markers, *i.e.* CRISPR oligotyping or carbohydrate metabolism, would be sufficient and among the best ways to differentiate the *L. rhamnosus* strains, providing a general approach to select the highest diversity in these and other bacterial species.

## Introduction

The current development and application of high-throughput sequencing technologies allow to intensively investigate complex microbial ecosystems, such as the human gastro-intestinal (GI) microbiota, consisting of over 3 million genes from mainly Gram-positive bacteria [1]–[4]. This and other metagenomic approaches obviate the necessity to culture bacterial isolates to comprehend the richness and the diversity of such ecosystem. However, detailed analysis at the strain level still requires isolation and growth of bacterial residents. Gram-positive lactobacilli are naturally found among ∼1000 phylotypes identified in the human intestinal tract [2], but only a fraction is represented in the present metagenomic sequences that derive from faecal samples. Lactobacilli mainly reside in the intestinal mucosa and were detected in the ileum metagenome [5], [6]. The limitations of metagenomic approach, *i.e.* sequencing depth, are well described and can be obviated by 16S rRNA sequencing and phylogenetic microarrays [7], [8]. Thus, Heilig *et al.* provided clear sequence-based evidence for the presence of *L. rhamnosus* related species in the human intestinal tract [9]. As a consequence of their interactions and ecological role in the human intestinal tract [10]–[13], lactobacilli are increasingly used in food production, food preservation and nutritional complement formulation [14]–[18]. One of the most used and documented lactobacilli marketed as a probiotic is *Lactobacillus rhamnosus* GG, which has been isolated from the human intestine and characterized extensively [19]–[21]. *L. rhamnosus* contains a 3.0-Mbp genome, among the largest of the lactic acid bacteria, and has the ability to persist in the human intestinal mucosa, as it produces pili that are decorated with the mucus-binding protein SpaC [22]–[26]. This significantly impacts the intestinal microbiota, *via* the displacement of pathogenic bacteria [27], modulation of epithelial barrier functions [28] and potential stimulation of the host immune system *via* bacteria-host surface molecule crosstalk [16], [29]–[31]. Since the interaction between host and bacteria has a pivotal role in the impact on the host, much research efforts are presently focused on characterizing the different interaction mechanisms, including the metabolic properties and host-signalling components of *L. rhamnosus* [30]. However, no studies have actually addressed the genomic diversity of the species *L. rhamnosus*, in spite of its extensive use in a variety of food products. While some *Lactobacillus* species have been found in only one dedicated niche, such as the milk-adapted *L. helveticus* [32], other lactobacilli such as *L. rhamnosus*, *L. casei* or *L. plantarum* have the capacity to colonize multiple habitats [15], [33]–[35]. More specifically, *L. rhamnosus* has been isolated from a large variety of ecological niches, *e.g.* human intestinal tract, vaginal cavity, oral cavity and cheese, exemplifying its remarkable ecological adaptability as a generalist [19], [36]–[39].

Genome sequence analysis of a number of lactobacilli revealed that their adaptation to diverse ecological niches is promoted by the acquisition of new genes by horizontal gene transfer and the decay or loss of non-essential genes [33], [35], [40], [41]. The domestication of some lactobacilli to the dairy environment is a typical example of a niche specialization, where milk-adapted strains have unusually high number of pseudogenes, reflected by the loss of metabolic pathways and transport systems that are non-essential in dairy niches rich in nutrients [40], [42]. In contrast, bacteria from the intestinal tract, a very dynamic habitat in terms of nutrient availability and bacterial population density, have broad metabolic capacities and lifestyle traits essential for survival, persistence and colonization in this niche, *e.g.* bile resistance [25], [43], anti-microbial activity [44], and mucus-binding pili expression [19]. In some cases, gene sets could even be specifically linked to a particular ecological niche, *i.e.* intestine *vs.* dairy environment, as reported for the related *L. acidophilus* and *L. helveticus* [40]. In *L. reuteri*, Frese and colleagues also demonstrated a host specialization between *L. reuteri* strains isolated from different vertebrates [45].

The present study of the species *L. rhamnosus* aimed at: *(a)* investigating the genomic diversity of the species and, *(b)* examining variable chromosomal regions associated with phenotypic and/or lifestyle traits found in *L. rhamnosus* isolates. Four complete *L. rhamnosus* genomes have been fully sequenced and assembled allowing us to have a glance at the diversity within the species [19], [46], [47]. In an effort to further comprehend the diversity and versatility of *L. rhamnosus* species, we sequenced and compared the genomes of 100 *Lactobacillus rhamnosus* strains that were isolated from different ecological niches and analyzed their phenotypes. This study represents the first large-scale genomic and functional analysis of *L. rhamnosus*, providing new insight in the genetics and lifestyle of this species that has a long history associated with human lifestyle and health.

## Results and Discussion

### General genomic features of the species *L. rhamnosus*

**Figure 1:**
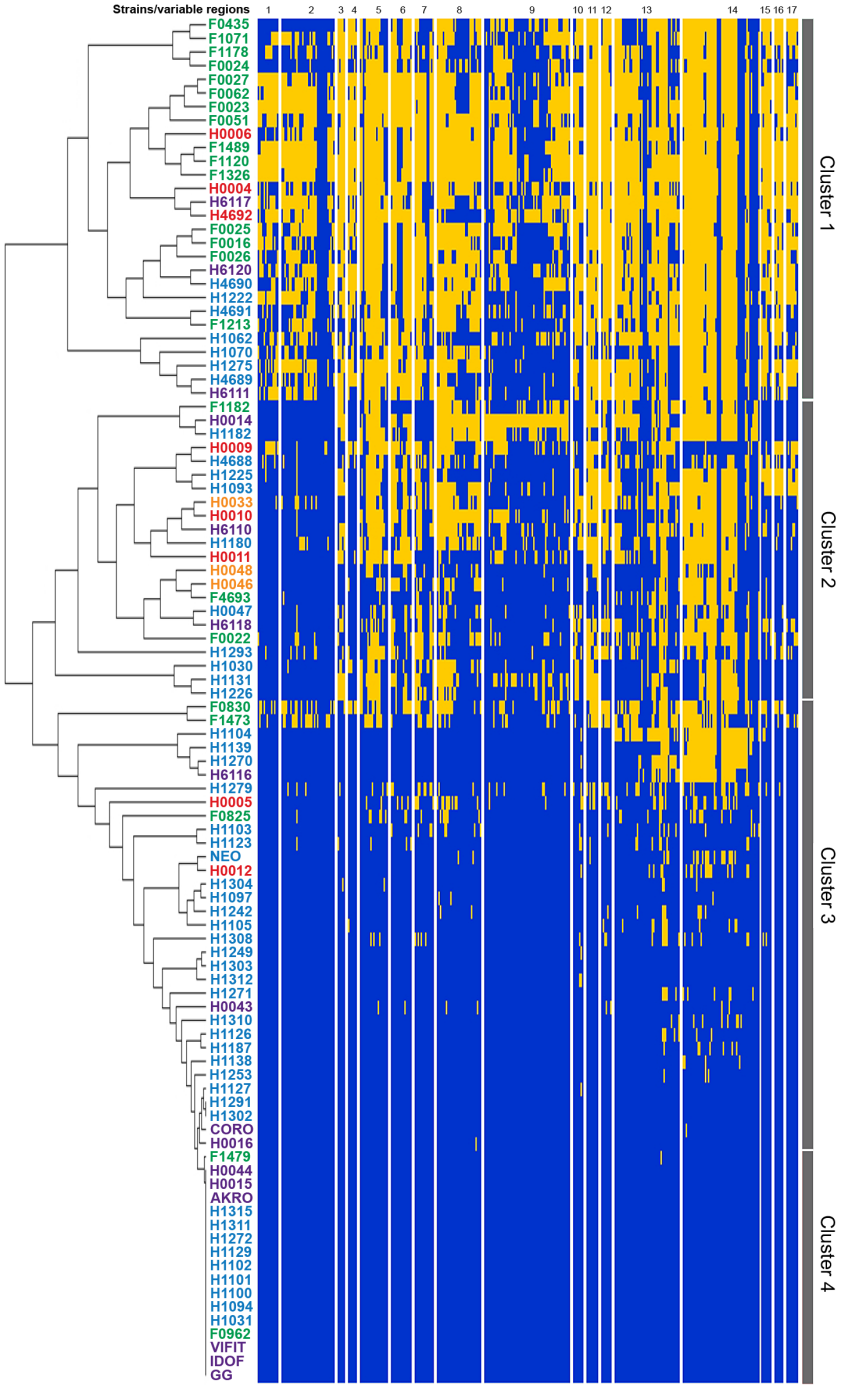
Analysis of genome diversity in *L. rhamnosus* by mapped SOLiD sequencing. The 100 *L. rhamnosus* strains were clustered using hierarchical clustering [78] based on their relative shared gene content with *L. rhamnosus* GG. Strain names were colour-coded as follows: green for dairy isolates, purple for intestinal isolates, orange for oral isolates, magenta for vaginal isolates and blue for clinical/other isolates. Four main groups or clusters were highlighted and numbered. The Figure 1 also shows the 17 variable chromosomal regions identified in GG, as further detailed in Table 1. Each row corresponds to one strain, and each column shows the genes in these variable regions, colour-coded as follows: blue for present and yellow for absent.

**Figure 2:**
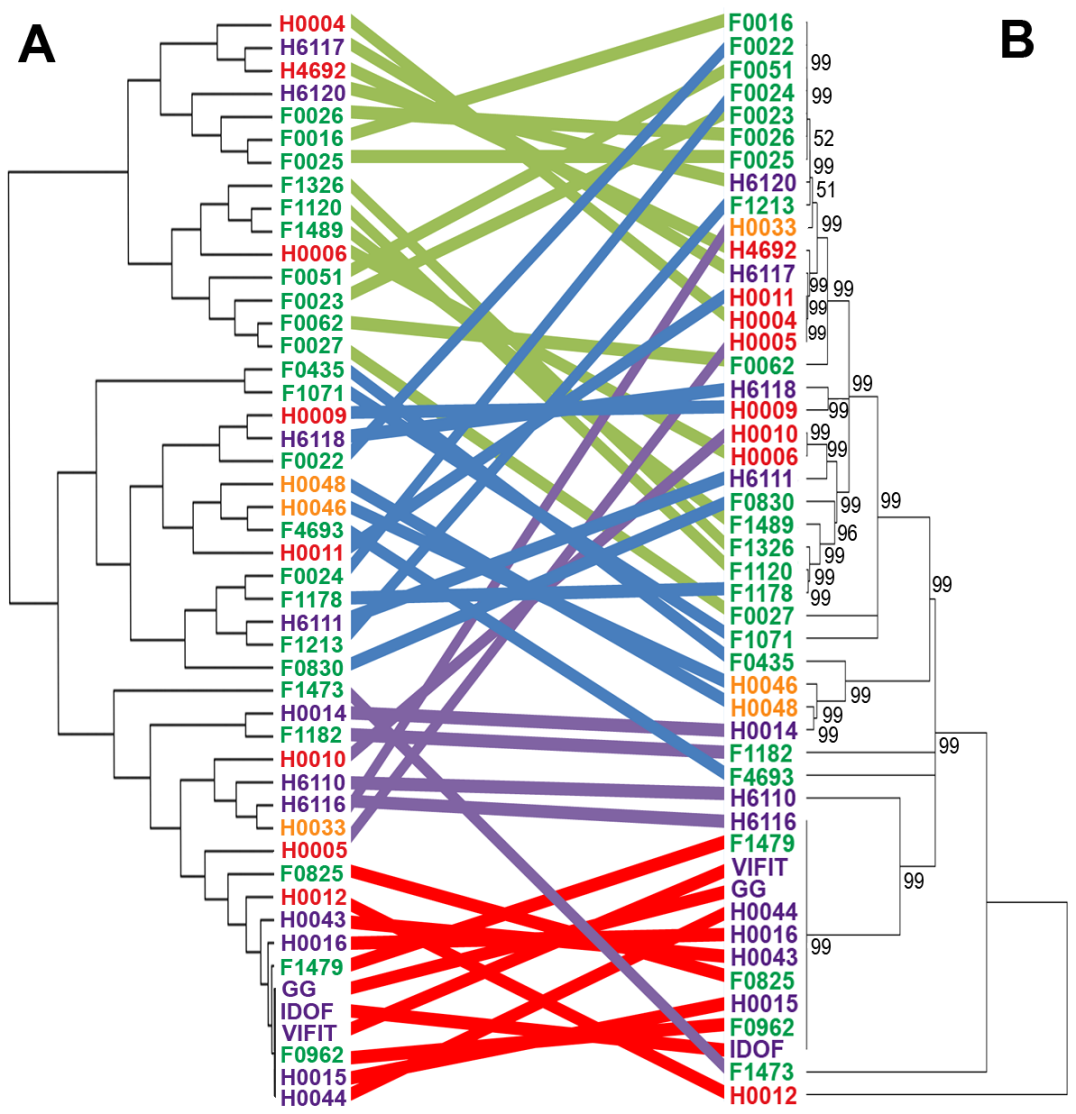
Comparison of hierarchical clustering and phylogenetic tree of a selected set of *L. rhamnosus* strains. Both hierarchical clustering (panel A) and phylogenetic tree (panel B) were performed on all *L. rhamnosus* strains, excluding isolates from unspecified or clinical origins. Coloured strings connecting the same strains of both trees aims at highlighting the degree of similarities between both tree methods [78], [79].

To comprehensively depict the phenotypic and genomic diversity of the *L. rhamnosus* species, 100 *L. rhamnosus* strains were isolated from a broad spectrum of ecological niches, *e.g.* 77 strains of various sites of the human body (oral cavity, vaginal cavity, blood and intestinal tract) and 23 strains of dairy origins, including artisanal cheeses and products marketed as probiotics (Table S1). The genomes of all strains were sequenced using the SOLiD sequencing technology and reads were mapped onto the *L. rhamnosus* GG chromosome [48]. This allowed detailed comparative genomic analysis and data mining as described in the Materials and Methods section. The number of shared genes between the 100 *L. rhamnosus* isolates and *L. rhamnosus* GG ranged from 2622/3016 (86.9%) to 3016/3016 (100%) genes with a median number of 2918/3016 (96.7%) genes. In terms of relative gene content, the dairy isolates significantly showed the most diversity with *L. rhamnosus* GG (average of 92.4%) than the human isolates (average of 96.04%, excluding clinical isolates), indicating that the dairy isolates are genetically most distant (*p*<0.001 between the two groups). It is noteworthy that 11 strains of human origin, 3 strains isolated from products marketed as probiotics, and only 1 strain isolated from artisanal cheese shared the complete set of 3016 genes present in *L. rhamnosus* GG. However, it has to be kept in mind that orthologous genes present in these isolates may carry mutations, *i.e.* single nucleotide polymorphisms, insertion and deletions that were not addressed in detail in this study. Therefore, the presence of a gene may not necessarily reflect its functionality, as observed within these 11 human strains, which showed significant phenotypic variations, *i.e.* sugar metabolism, indicating that these strains are not *L. rhamnosus* GG (see below). Moreover, strain-specific genes are likely to be present in these isolates, conferring additional phenotypic traits not present in *L. rhamnosus* GG. Based on comparative gene content, the hierarchical clustering of the *L. rhamnosus* species resulted in four distinct clusters (Figure 1). Remarkably, most dairy strains were found to belong to the cluster 1 and show marked differences with other clusters. In contrast, intestinal isolates, including *L. rhamnosus* strains marketed as probiotics shared similarities with other human isolates (Figures 1 and 2). This is in line with the hypothesis that the genomes of probiotic-marketed strains still reflect their adaptation to their original isolation source, *i.e.* the human intestinal tract [19]. The distribution of the clinical isolates all across the clustering rather reflects their original ecological niche than their isolation source, since infections are extremely rare events and evolutionary dead ends. The clusters 3 and 4 consist predominantly of *L. rhamnosus* strains closely related to *L. rhamnosus* GG (Figure 1). In Figure 2, comparison of hierarchical clustering and phylogenetic tree shows some degree of conservation in the grouping of the strains. The phylogenetic tree reflects slow evolution within the genome, *i.e.* point mutations, whereas the genomic tree (or hierarchical clustering) describes major genetic re-arrangement events, *i.e.* insertions or deletions. Hierarchical clustering therefore shows more recent chromosomal changes, where recombination events contribute to the diversity of the species. Similar differences have been observed in other species, such as *L. casei* [41].

Based on the 100 mapped genomes, we defined a set of all orthologous genes that are shared by all *L. rhamnosus* strains. We observed that the shared gene set (core) of the *L. rhamnosus* species consists of 2419 genes, which represents 80.2% of *L. rhamnosus* GG genome. The larger the set of strains used, the smaller the core genome becomes, a trend observed in other genomes as well, such as the core-genome of *Streptococcus agalactiae* and other bacterial species [49], [50]. However, the size of the core genome remained stable above ∼20 genomes (data not shown). The full comparative genomic results are shown in Tables S2 and S3. Although the characterization of *L. rhamnosus* pan-genome would bring further insights into the species, we did not address it in the present study, as this would require complementary sequencing techniques. Further deep and full-coverage sequence analysis of a selected subset of heterogeneous *L. rhamnosus* strains is now on-going to report the pan-genome of the species (data not shown). The initial read mapping to the reference genome *L. rhamnosus* strain GG clearly give a GG-centric view of the genome diversity within the species. However, the additional read mapping to the dairy strain LC705 of a selected set of *L. rhamnosus* strains revealed a similar clustering as in Figures 1 and 2 (data not shown). This suggests that the use of one strain or another as a reference does not impact on the hierarchical clustering of the isolates and also supports the validity of the experimental design approach chosen in the present study.

**Figure 3:**
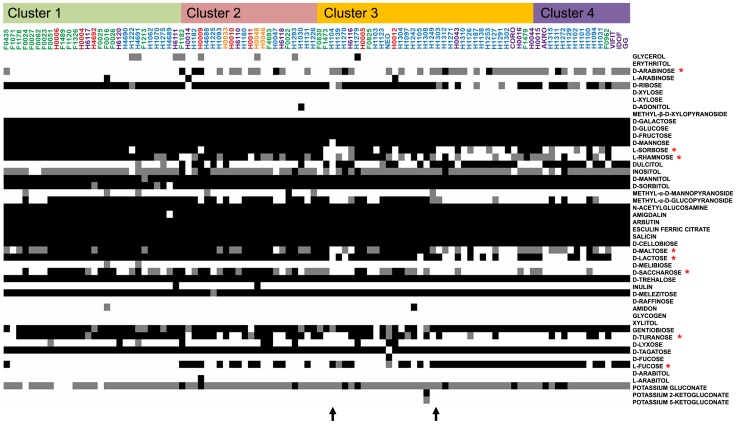
API 50CH fermentative profile of *L. rhamnosus* strains. Fermentation ability is indicated in black for positive, grey for partially positive and white for negative. Strains are organized according to their genetic relatedness as defined in the hierarchical clustering and coloured according to their respective niche/origin (Figure 1). Carbohydrates of interest are marked by a red asterisk. Black arrows show fermentative profile shifts among *L. rhamnosus* strains.

The distribution of Clusters of Orthologous Groups of proteins (COG) was determined for *L. rhamnosus* GG genome, the *L. rhamnosus* core-genome and the non-core gene set (Figure S1). Although no major differences in the relative COG distribution between the different subsets were found, it is noteworthy that 87 *L. rhamnosus* GG genes (30.2%) out of 288 genes assigned to the COG ‘Carbohydrate transport and metabolism’ are not in the estimated core genome and are predicted to encode mostly phosphotransferase system (PTS) and other sugar transport systems, possibly essential for the persistence in the intestinal tract. These genes were located in highly variable regions of the *L. rhamnosus* genome, reflecting the metabolic diversity of this species (Figure 3). The 17 most variable chromosomal regions include all genomic islands (GIs), typically rich in transposases and other mobile genetic elements (Figure 1 and Table 1). In *L. rhamnosus* GG, 5 GIs had previously been identified [19]. The presence of these genomic islands greatly varies among strains of the species *L. rhamnosus*, as observed previously for the strains LC705 and GG [19]. This suggests that horizontal gene transfer events have contributed significantly to the diversity of the *L. rhamnosus* species. The GIs identified here were associated with specific biological functions, including interaction and signalling with the host, optimal use of available nutrients and protection against autochthonous phages and mobile genetic elements. Hence they may be considered as lifestyle islands, as their predicted function may specifically contribute to the persistence and colonization in intestinal and other habitats. Other variable regions consisted mostly of transposases and conserved proteins with no clear function and were not further addressed (Figure S2).

**Table 1:** Features of the variable chromosomal regions found in *L. rhamnosus*.

Region	Genes	GI	IS	Main genetic features of the region
1	LGG_00170–LGG_00177	-	-	taurine ABC transporter, conserved protein, transcriptional regulator
2	LGG_00278–LGG_00283	-	-	rhamnosyl PTS, rhamnosyltransferase
3	LGG_00341–LGG_00347	-	-	galactitol PTS, conserved protein
4	LGG_00376–LGG_00427	1	2 IS	transcriptional regulator, hypothetical protein, fructose PTS, lactose PTS, mannose PTS, conserved protein,
5	LGG_00438–LGG_00481	2	11 IS	conserved protein, SpaCBA pili cluster, transcriptional regulator, ABC transporter
6	LGG_00511–LGG_00517	-	2 IS	ABC transporter, conserved protein
7	LGG_00559–LGG_00566	-	-	conserved protein, transporter, sugar phosphate isomerase
8	LGG_01023–LGG_01029	-	3 IS	restriction/modification enzymes
9	LGG_01086–LGG_01143	3	-	conserved protein, phage-related protein
10	LGG_01515–LGG_01544	4	1 IS	phage-related protein, conserved protein
11	LGG_01955–LGG_01967	-	5 IS	conserved protein
12	LGG_01990–LGG_02003	-	1 IS	conserved protein, UDP-N-acetylglucosamine 2-epimerase, lysozyme
13	LGG_02038–LGG_02056	5	1 IS	EPS cluster
14	LGG_02199–LGG_02204	-	-	CRISPR locus
15	LGG_02610–LGG_02614	-	-	ABC transporter, conserved protein
16	LGG_02651–LGG_02686	-	1 IS	fucose transporter, conserved protein, transcriptional regulator
17	LGG_02742–LGG_02755	-	1 IS	conserved protein, fructose-bisphosphate aldolase, mannose/fructose/lactose PTS, galactitol PTS

### Metabolic islands, carbohydrate transport and metabolism and niches

Comparative genomic analysis of the 100 strains revealed the loss of genes encoding various carbohydrate PTS system and metabolism-associated proteins compared to *L. rhamnosus* GG. To study the impact of these genomic characteristics, the metabolic capability to utilize different carbon sources was investigated. Carbohydrate utilization profiling showed that most *L. rhamnosus* strains use a large range of simple and complex carbohydrates (Figure 3). However, some differences may reflect their genomic diversity and also at some extent how they evolved in different ecological niches, by the acquisition or the loss of metabolic-associated genes. The ability to utilize carbohydrates mostly relies on the presence of functional transporter machinery and intact metabolic pathways. The clustering of *L. rhamnosus* strains (Figure 3) revealed strong associations between genome diversity, carbohydrate metabolism and their origins. Typically, strains belonging to cluster 4 utilize D-arabinose, dulcitol and L-fucose, whereas other strains lost these functions but possess the ability to ferment L-sorbose, D-maltose, D-lactose, D-turanose, methyl-α-D-glucopyranoside, L-rhamnose and D-saccharose (Figure 3). Hence, we detail the differences in carbohydrate utilization within the *L. rhamnosus* species below.

The genome of *L. rhamnosus* GG harbors a tagatose-6-phosphate pathway (*lacABCD*) and a lactose PTS (*lacFEG*) but the antiterminator *lacT* and the phospho-β-galactosidase encoding *lacG* genes are altered and non-functional, preventing GG from metabolizing D-lactose [19]. Strains belonging to the cluster 4 also show a poor ability or incapacity to use D-lactose, whereas other isolates, including most dairy ones utilize this disaccharide, which is found in milk and milk-derived products. We propose that the *lacT* and *lacG* genes have been kept intact in these strains, as lactose represents an important carbon source and provides a real benefit for *L. rhamnosus* strains residing in dairy niches. The maltose locus was predicted to be non-functional in *L. rhamnosus* GG due to the insertion of a conserved gene (LGG_00950) between genes encoding the maltose-specific *malEFGK* transporter and the hydrolase (LGG_0954-LGG_0951 and LGG_00949, respectively) [19]. Similarly, we found that most *L. rhamnosus* strains unable to use maltose also contained a maltose locus disrupted by LGG_00950. In contrast, the majority of strains belonging to other sublineage contained an intact maltose locus and were able to utilize maltose, indicating that the insertional inactivation by LGG_00950 may have played a significant role in *L. rhamnosus* species ecology. Comparative genome sequencing of *L. rhamnosus* GG also showed that the rhamnose locus is altered: the galactitol-specific *gatABCD* PTS and a DeoR transcriptional regulator are missing while the *rhaB* gene is duplicated, possibly explaining the inability to use rhamnose compared to some other *L. rhamnosus* strains, such as LC705 [19]. Combination of the genomic and metabolic data indicates that most strains of the cluster 4 similarly contain a defective rhamnose locus. It is noteworthy that 74% of all isolates can partially or fully utilized L-rhamnose, a carbohydrate from which the species name derives. In contrast, fucosylated compounds such as human mucin and other glycoproteins play an important role in the human gut ecology, as a carbon source for intestinal bacterial species [30]. Close inspection of the L-fucose metabolism revealed that a large number of dairy-associated strains are unable to use L-fucose due to the lack of one or multiple genes required to transport and to metabolize L-fucose: the *fucU* and *fucI* isomerases, *fcsR* fucose operon repressor and α-L-fucosidase (LGG_02652). Most strains closely related to *L. rhamnosus* GG retained the capacity to use L-fucose, whereas dairy strains lost this ability, since L-fucose is not as abundant in bovine milk. Dulcitol, a polyol also known as galactitol, is used by the cluster 4 (Figure 3). In some strains unable to use dulcitol, the function loss was associated with the lack of an intact *gatABC* PTS system. Other carbohydrates such as turanose and sorbose were not metabolized by strains related to GG (Figure 3). In *L. rhamnosus* LC705, an intact sorbose *sorABCDEFGR* locus is present, explaining its ability to utilize sorbose, whereas *L. rhamnosus* GG lacks such machinery [19]. *L. rhamnosus* strains with similar capabilities may therefore possess an intact sorbose locus. Remarkably, the strains from the cluster 1 present a similar metabolic profile as the industrial dairy strain *L. rhamnosus* LC705 [19]. This suggests that dairy-related strains characterized in the present study underwent similar niche adaptation as LC705 in terms of acquisition, decay or loss of genes.

### Diversity of the Clustered Regularly Interspaced Short Palindromic Repeats-Cas system: A spacer oligotyping analysis

**Figure 4:**
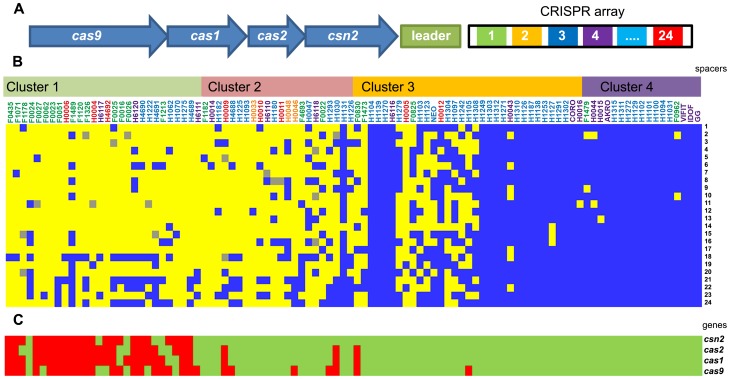
CRISPR spacer oligotyping and CRISPR-associated protein diversity in *L. rhamnosus* species. Panel (A) illustrates the genetic organization of the CRISPR system and its associated genes in *L. rhamnosus* GG. Panel (B) shows the conservation (blue), the partial conservation (grey) or the absence (yellow) of *L. rhamnosus* GG spacers. The presence (green) or the absence (red) of the *cas* genes is also indicated in Panel (C). Strains are organized according to their genetic relatedness defined in Figure 1.

**Figure 5:**
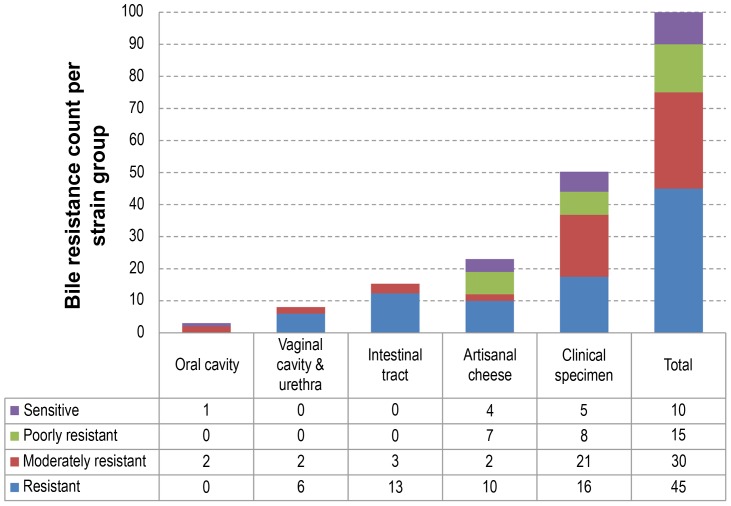
Bile resistance distribution among the different niches or groups. Strains were classified as resistant, moderately resistant, poorly resistant or sensitive to bile salts. The table below the histogram details the bile resistance distribution of strains in each niche or group.

CRISPR (clustered regularly interspaced short palindromic repeats) loci are present in a large number of prokaryote genomes [51], playing an important role in controlling horizontal gene transfer. It has been well established that some bacteria acquired the CRISPR-Cas system as a protection/immunization system against plasmid conjugation and phage predation [52]–[55]. The CRISPR-Cas system usually consists of a leader sequence, an array of CRISPRs interspaced by spacers and a *cas* gene cluster encoding the Cas protein complex (Figure 4A) [56]. The role and mechanistic of the CRISPR-Cas system in bacterial species have been extensively studied and indicate that the spacer sequences can be considered as a signature of past exposure to exogenous DNA [57]. *L. rhamnosus* GG has a single Type II-A CRISPR-Cas locus, consisting of 4 *cas* genes and one CRISPR array containing 24 spacers [19]. To determine whether the CRISPR sequences could be used as an indicator of a specific niche, we determined their diversity and the presence of the *cas* genes. CRISPR genotyping has been previously developed for epidemiological purposes and strain differentiation for *Mycobacterium tuberculosis* [58], enterohemorrhagic *Escherichia coli* [59] and *Salmonella enterica* [60]. We were able to generate a CRISPR profile (based on spacer oligotyping) for each strain and this revealed a high degree of diversity among the various strains (Figure 4B, C). Remarkably, all strains from cluster 4 were sharing a comparable CRISPR spacer set, whereas the genetically more distant *L. rhamnosus* strains were only harbouring few of the spacers found in *L. rhamnosus* GG and a poor conservation of the *cas* genes. The overall CRISPR-Cas typing analysis showed that strains from the same sublineage mostly shared identical CRISPR-Cas loci. Interestingly, strains H1093 and H4692 did not have any of *L. rhamnosus* GG spacers but some of the *cas* genes remained present, whilst strain H1275 lacked the entire CRISPR-Cas locus. It has to be kept in mind that only sequences homologous to the CRISPR-Cas locus from strain *L. rhamnosus* were identified, allowing the possibility that additional spacers, *cas* genes or even additional CRISPR loci may be present. To determine the function of the CRISPR-Cas system in protecting *L. rhamnosus* from exogenous DNA, blastn searches on all 24 spacers were performed against virus and plasmid database at GenBank. Out of 24 spacers, 11 spacer sequences showed substantial sequence identity with plasmid or phage sequences (Table S4). Eight spacer sequences fully or partially matched known bacteriophages genomes: *L. rhamnosus* phage Lc-Nu, *L. casei* phage φ AT3, *L. casei* phage Lrm1, *L. casei* phage A2 and *L. casei* phage PL-1. The identified CRISPR spacers thus belonged to phages from *L. rhamnosus* strains or closely related bacterial species, *i.e. L. casei*, highlighting the role of the CRISPR-Cas system as an immunity system against phage predation. Some spacers (4, 12, 18, 21 and 22) have multiple phage hits, showing that the corresponding phage genomes share the same region, preventing us to predict from which bacteriophage these particular spacers were acquired. One match for plasmids was also found: the conjugative plasmid pSB102. The data also indicates that the CRISPR-Cas system may play a role in the *L. rhamnosus* species diversity by controlling horizontal gene transfer and providing phage resistance, thereby contributing to diversification of the species. Our data also showed that the degree of CRISPR diversity correlated with the genomic clustering of the 100 isolates and at some extent with their ecological niche (Figure 5). Most dairy isolates shared only 6–7 spacers with *L. rhamnosus* GG, indicating that the variety and the exposure to phages and other mobile genetic elements differ in each habitat, *i.e.* the intestinal tract and cheese. We anticipate that some of the dairy strains may have an entirely different set of CRISPR sequences, representative of their own habitat and possibly additional CRISPR-Cas Types, as seen across the lactic acid bacteria [61].

### Bile resistance, a persistence trait

All 100 *L. rhamnosus* isolates were tested for resistance to bile salts, a property that is usually associated with the intestinal tract environment (Figure 5). A majority of *L. rhamnosus* strains were bile resistant (45% resistant and 30% moderately resistant) and different bile resistance profiles were observed in each niche (Figure 5). No clear association could be seen when combining the bile salt resistance data with the hierarchical clustering. A similar distribution was observed in strains isolated from clinical specimens and cheeses, even though a slightly higher proportion of bile salt-sensitive strains could be observed in the cheese isolate group. As expected, all strains from the human intestinal tract were resistant to bile salts, illustrating that such trait is essential for persisting in the intestinal tract. All vaginal isolates also showed bile resistance, suggesting that *L. rhamnosus* strains of the colonic microbiota may possibly have colonized the vaginal cavity as previously reported [62]. The low number of isolates from oral cavities (*n* = 3) did not allow us to draw any conclusions, but revealed a different profile in terms of bile sensitivity. One of the hyper-variable regions in GG contained genes encoding the taurine transport system *tauABC*, potentially involved in the bile salt conjugation. Nine out of 25 bile-sensitive strains had a defective *tauABC* locus, suggesting that the *tauABC* locus may affect the bile sensitivity of these strains although most likely additional genes are involved.

### Pilosotype diversity

**Figure 6:**
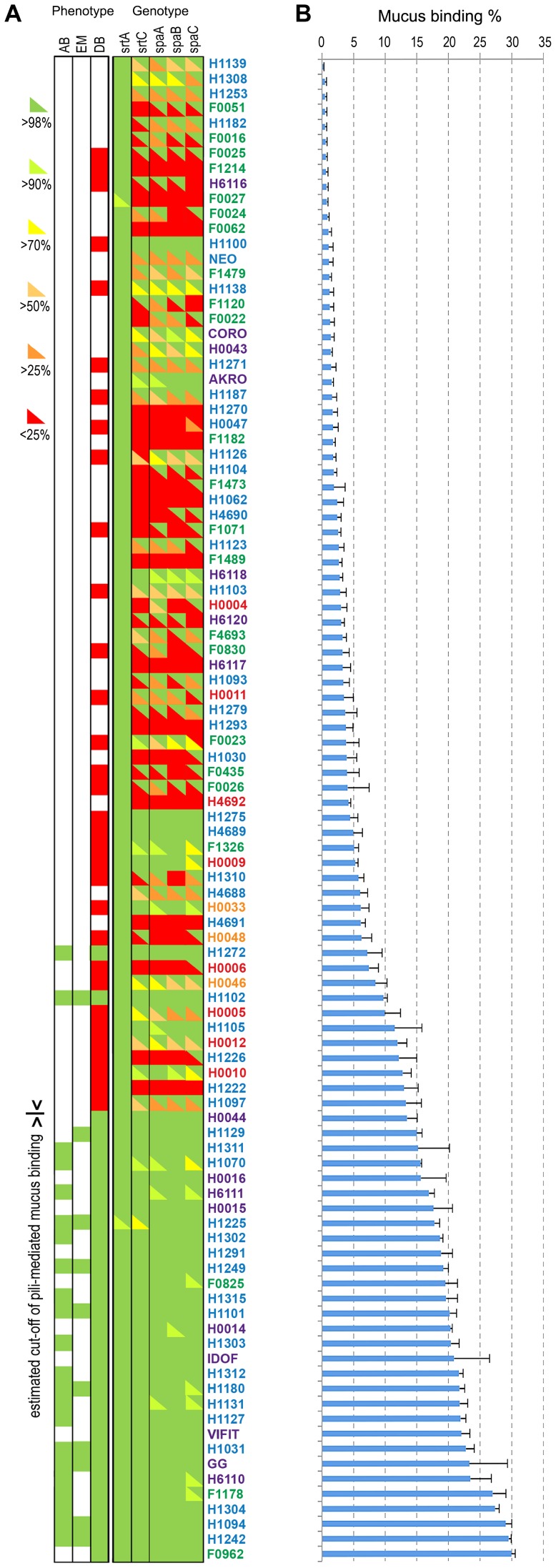
Mucus adhesion and SpaCBA pili gene diversity among *L. rhamnosus*. Panel (A) shows the genotype and phenotype of all strains. Based on our genomic analysis, pilin and sortase genes were assigned as present (green) or divergent (red). Sequences of corresponding genes were further analyzed using blastx. The sequence identity was shown by an upper triangle superposed to the SOLiD genomic data, where the colour gradient corresponds to the identity percentage to GG pili genes. We also indicated if the strains were tested by immunoblotting analysis (DB), electron microscopy (EM) or *in vitro* competitive binding assay (AB). Green is for pili positive and red for pili negative. Panel (B) shows the human mucus binding ability (%) of all *L. rhamnosus* isolates ranked from the lowest to the highest mucus binder.

Pili in *L. rhamnosus* strains play a significant role in terms of interaction, colonization, persistence and potential signalling in the human intestinal tract [19]–[21]. The *spaCBA* pili gene cluster is flanked by numerous IS elements, suggesting that *L. rhamnosus* might have acquired the *spaCBA* pili gene cluster by horizontal gene transfer [41], [63], where the integration of the *iso-IS*30 element had constituted a promoter that allowed the expression of the pili genes in *L. rhamnosus* GG [63]. It also indicates that this IS element-rich chromosomal region may be subject to important genetic recombination events within the species [19], [64]. Hence, we examined the pili diversity among all 100 isolates, providing a detailed picture on the conservation of the pili genes in each strain, since as little as one mutation is potentially sufficient to prevent the pili production or to affect the mucus binding abilities (Figure 6). Moreover, to support the genomic data, we investigated the mucus adhesion abilities of all *L. rhamnosus* isolates and also verified the presence of pili in a number of these strains by immunoblotting analysis (*n* = 64), transmission electron microscopy (*n* = 10) and *in vitro* blocking mucus binding assays (*n* = 22) (Figures 6, S3 and S4). The mucus binding capacity ranged from 0.05% to 29.9% in all tested strains and was clearly correlated with the presence of a functional SpaCBA pili gene cluster, as shown at both genomic and phenotypic levels (Figure 6). To further demonstrate that the mucus binding capacity of these strains was mediated by SpaCBA pili, we performed *in vitro* blocking mucus binding assays on 22 SpaCBA-positive isolates using SpaC anti-serum as previously described (Figures 6 and S3) [19]. In all 22 strains tested, the addition of SpaC anti-serum significantly reduced mucus binding, indicating that the SpaCBA pili has a major role in the interaction between *L. rhamnosus* and the human intestinal mucus. Remarkably, some strains displayed significant mucus-binding capacity but lacked the canonical SpaCBA pili structures, suggesting that alternative interaction players are involved. The genes encoding the SpaCBA pili of some strains such as H1242, H1304, F1178 and H6110 are highly conserved but, however, with some subtle sequence differences. We propose that the sequence polymorphism of the pili genes in these strains might modulate mucus binding capacity or affinity. Alternatively, we cannot rule out that additional strain-specific traits might be involved in the mucus binding, especially in strain F1178 where the residual binding in the presence of SpaC anti-serum still remained high (Figure S3). In contrast, strains with poor mucus-binding abilities appeared to have some remnants of pili genes in a more or less decayed form (Figure 6). In strains H1275, H4689 and H1100, the *spaCBA* pili gene cluster is highly conserved (>98%), but show a very poor binding, indicating that the pili production may be impaired by critical mutation(s) or a defective promoter.

The *L. rhamnosus* strains were further classified according to two main criteria, *i.e.* their ecological niche and their pilosotype, defined as the presence of pili genes that encode functional pili (Table 2). The results indicate that the production of functional SpaCBA pili was significantly more prevalent in human isolates (40.2% or 31/77) than in dairy isolates (13% or 3/23). This suggests that the lack of the SpaCBA pili gene cluster in most dairy strains reflects a possible niche specialization to a habitat where pili structures are not essential and do not bring any benefit for persistence and colonization. Among all niche groups, the intestinal strains are the most prevalent group to produce SpaCBA pili, which would confer the ability to efficiently colonize and persist in the intestinal tract. In contrast, none of the strains originated from the oral and vaginal cavities produces functional pili, indicating that such trait may not be required in these two ecological niches. Our observations support the hypothesis that the human-mucus binding properties of pili may constitute an advantage to the lactobacilli to persist in the intestinal tract, but may be lost in strains evolving in other ecological niches, such as dairy products, through the decay or loss of the non-essential SpaCBA pili gene cluster.

**Table 2:** Pilosotype distribution in our *L. rhamnosus* collection.

Source of isolation	SpaCBA positive	SpaCBA negative	Total	% SpaCBA
**Human**	**31**	**46**	**77**	**40.2**
Blood	14	17	31	45.1
Vaginal cavity/urethra	0	8	8	0
Oral Cavity	0	3	3	0
Intestinal tract	9	7	16	56.2
Others	8	11	19	42.1
**Dairy products**	**3**	**20**	**23**	**13.0**
Parmigiano Regiano cheese	3	9	12	25.0
Pecorino cheese	0	9	9	0
Other cheeses	0	2	2	0

The table describes the niches or isolation sources, the number of strains per group and their pilosotype, *i.e.* the presence of an intact and functional SpaCBA pili cluster as determined in Figure 6. Probiotic strains GG, VIFIT, IDOF, AK-RO and CO-RO were classified as intestinal isolates. The group ‘Others’ contained strains of unspecified origins (clinical specimens) or from minor isolation source (*n*<2), *i.e.* hip punction or pus.

### Cross-talk between *L. rhamnosus* and intestinal cells

Due to the intimate interaction between *L. rhamnosus* and the intestinal mucosa [30], we studied the potential signalling pathways that could be triggered by the *L. rhamnosus* strains. This was realized by determining the signal transduction in intestinal epithelial cells *via* Toll-like Receptors (TLRs) TLR-2, TLR-4 and TLR-5. All 100 isolates were tested for signallings *via* TLR-4 and TLR-5 receptors, but no significant responses were observed, which is in agreement with the identified ligands for these two TLRs, *i.e.* lipopolysaccharides and flagellins respectively (data not shown). Clearly, *L. rhamnosus*-host signallings are mediated through different receptors. Signalling *via* the TLR-2 receptor in *L. rhamnosus* species was observed and greatly varied among isolates (Figure S5). More than half of the isolates mediated a TLR-2 response very similar to the level observed for strain GG after 1 h (fold-induction of ∼1.5). Six strains (H6111, H0009, H4692, H1311, H1226 and H1131) triggered a stronger TLR-2 response in this assay system. We did not determine the nature of the ligand recognized by TLR2 but assume in analogy with what has been found in *L. rhamnosus* GG that the signalling might be mediated by the lipoteichoic acids [65]. The levels of TLR2 signalling could not be correlated with any other traits, such as EPS production, pili production or the presence of other membrane-associated proteins. No links between the TLR2 response, hierarchical clustering and ecological niches of the various strains were either identified. This suggests that the TLR-2 response triggered by *L. rhamnosus* does not reflect its adaptation to one particular niche, but is rather a trait acquired, maintained, altered or exacerbated by other factors that remains yet to be identified.

### *L. rhamnosus vs.* other bacterial populations

*L. rhamnosus* isolates have been isolated from various ecological habitats, showing its large ecological versatility. Niche-specialized strains have developed distinctive metabolic traits, phage resistance system, stress-resistance mechanisms and colonization traits to efficiently persist in an ecological habitat. However, the microbiota of habitats such as the human intestinal tract or the vaginal cavity are rich and complex, consisting of many phylotypes [2], [66]. *L. rhamnosus* strains may therefore compete with other bacterial species by producing bacteriocins that prevent growth of other bacterial populations. In contrast, the diversity and richness of the microbiota in dairy products is much lower, suggesting less competition [67]. When testing the anti-microbial activity of 92 *L. rhamnosus* strains, we found that most strains displayed anti-microbial activity against pathogens *E. coli*, *Yersinia enterocolitica* and *Listeria monocytogenes* at different pH (Figure S6). This is in line with previous studies on *L. rhamnosus* anti-microbial activity [44], [68], [69]. Remarkably, most dairy isolates shared comparable anti-microbial capabilities and clustered together, *e.g.* poor anti-microbial activity against *E. coli* and, to a lesser degree, against *L. monocytogenes*. The human strains displayed a differential spectrum and level of antimicrobial activity against the three human pathogens tested than most dairy strains. This illustrates the fitness of human isolates to compete with other bacteria potentially present in the human body cavities. In contrast, a high proportion of dairy isolates seems to have lost the ability to produce antimicrobial compounds against these three human pathogens, suggesting that such trait might not be essential in an environment with a lower and different microbiota diversity than in human body cavities. It, however, does not imply that those isolates do not produce antimicrobial compounds active against other pathogens more prevalent in their respective niche.

### Concluding remarks: Strain diversity and niche adaptation

**Figure 7:**
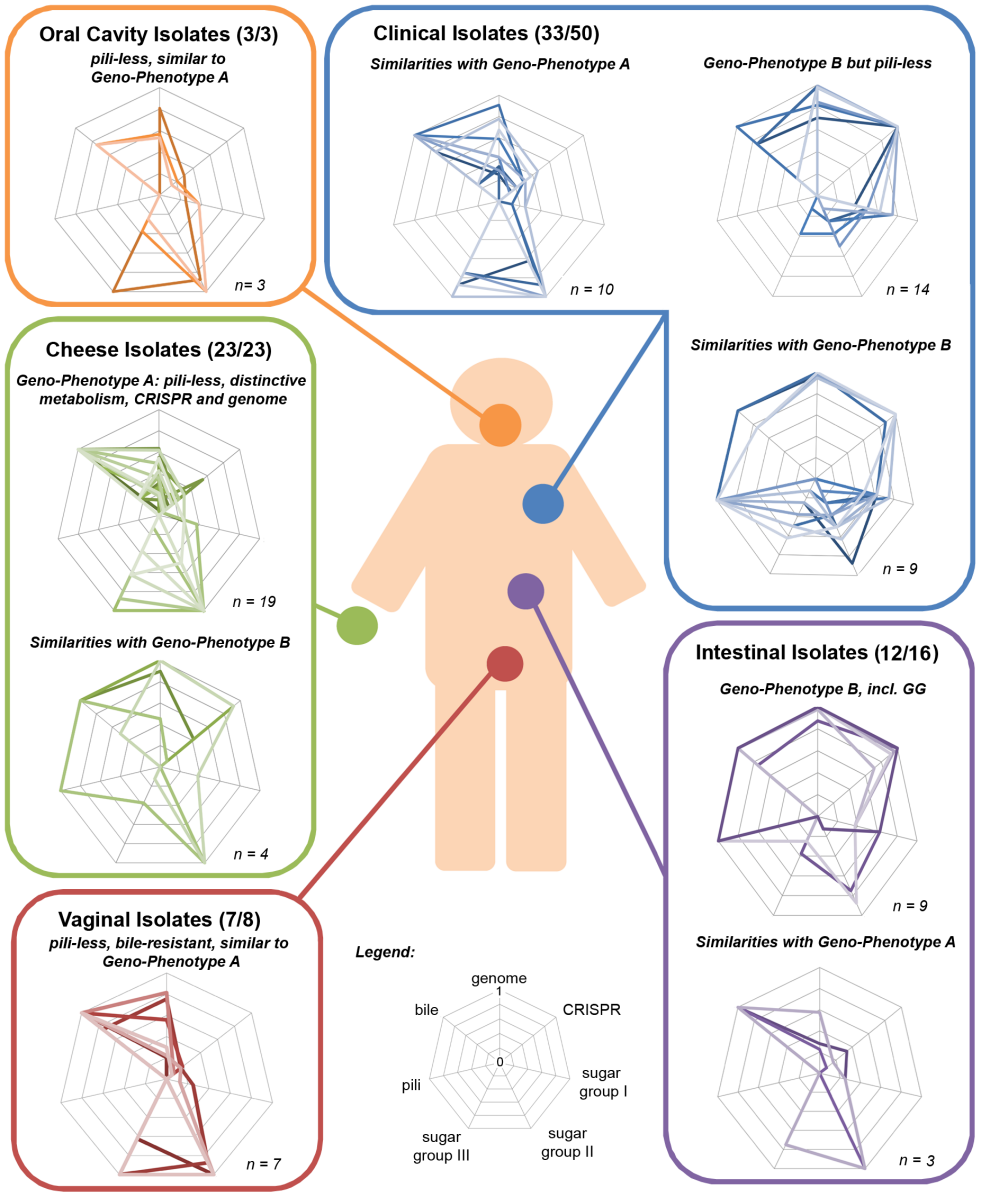
Anthropocentric view of the *L. rhamnosus* species. The interactions between *L. rhamnosus* and the human cavities are frequent and occur in various contexts, *i.e.* consumption of food products (common scenario) or development of bacteremia (rare event). For each niche or isolation source, the strains were grouped according to their geno-phenotype (radar plot). The geno-phenotype is based on the scoring of distinctive genetic and phenotypic traits measured in this study, *i.e.* gene-content, CRISPR oligotype, bile resistance, pilosotype, sugar group I (dulcitol, D-arabinose and L-fucose), sugar group II (D-saccharose, D-maltose, methyl-α-D-glucopyranoside and D-turanose) and sugar group III(L-rhamnose, L-sorbose, D-ribose and D-lactose). The distinction between the two main geno-phenotypes mostly relies on gene acquisition and loss, point mutations, genetic reorganization that possibly reflect strain adaptation to an ecological niche.

The species *L. rhamnosus* has been isolated in various dairy products and human body cavities, highlighting the close association and frequent interactions between *L. rhamnosus* and the human body. The analysis of the genomes and phenotypes of 100 strains of the species *L. rhamnosus* provides then a wealth of information with respect to the traits that are beneficial or essential in different ecological niches and, also allowed us to depict in details the species from an anthropocentric perspective. As expected, close inspection of the hierarchical clustering of the 100 *L. rhamnosu*s strains showed that this can be paralleled to some extent by clustering of phenotypic data, *i.e.* carbohydrate metabolism, antagonistic activity, CRISPR oligotyping, bile salt resistance or pilosotype. Interestingly, the integration of both phenotypic and genomic data of each strain revealed the presence of two prevailing geno-phenotypes called A & B in the *L. rhamnosus* species (Figure 7). The strains belonging to the geno-phenotype A are characterized by a lack of SpaCBA pili, a different carbohydrate metabolism (D-lactose, D-maltose and L-rhamnose) and a distinct CRISPR system profile, indicative a possible adaptation to dairy-like environment. In contrast, the geno-phenotype B depicts strains with a specific set of lifestyle traits that would confer them adequate fitness to the intestinal tract, such as bile resistance, pili production and L-fucose utilization. The geno-phenotype B showed a high similarity with *L. rhamnosus* GG in terms of genomes and phenotypes.

The geno-phenotype A is prevalent in the cheese group, indicating their adaptation to the dairy environment. The PTS and metabolic-related genes non-essential in dairy products were lost or decayed, *i.e.* loss of L-fucose utilization. In parallel, we hypothesized that additional functions were acquired possibly through horizontal gene transfers, genetic mobile elements or plasmids, *i.e.* the ability to use lactose, a major carbon source in milk-derivative products. The loss of pili in these dairy strains is another characteristic example of a trait lost during niche-adaptation, where the absence of mucosa surfaces is reflected by the decay or complete loss of non-essential pili. In dairy niches, phage predation is ubiquitous as showed in many studies of lactic acid bacteria [70], [71] and the CRISPR system might evolve by the acquisition of spacers representative of phages or plasmids of a particular niche. This is the case as the CRISPR locus profile between both geno-phenotypes differs considerably.

Interestingly, *L. rhamnosus* from the vaginal cavity and urethra have a geno-phenotype A, which is in agreement with previous studies showing that the rectal microbiota is a potential reservoir of bacteria that may colonize the vaginal cavity [62]. This also suggests that the intestinal isolates (geno-phenotype A) may be more adapted to the vaginal environment, possibly due to their distinct metabolic abilities. This remains speculative, as at individual level, we do not know which *L. rhamnosus* strains these women possibly have in the intestinal tract. Interestingly, the oral isolates also possess a geno-phenotype A. Due to the low number of strains, it is difficult to draw any definitive conclusions for the oral group. However, the prevalence of the geno-phenotype A in these three niches highlights a close link between them, indicating that the geno-phenotype A strains may likely originate from either dairy products but also oral or vaginal cavities.

Both geno-phenotypes A and B were found among the intestinal isolates (Figure 7). We proposed that the geno-phenotype A strains were likely introduced in the intestinal tract *via* consumption of foods. Bile resistant and with different metabolic capabilities, they are able to survive in the intestinal tract but may not be able to compete with other autochthonous intestinal bacteria to colonize the intestinal tract, *i.e.* lack of mucus-binding pili. This would indicate the most of these isolates are *transient* in the intestinal tract and further eliminated along with the faecal material. Other *L. rhamnosus* dairy isolates that are bile sensitive may also be introduced in the gastro-intestinal tract through the diet but cannot survive the intestinal conditions. On the other hand, geno-phenotype B strains are likely to be autochonous, as they possess phenotypic traits, promoting resistance and persistence in the human intestinal gut. Still, we cannot exclude the hypothesis that geno-phenotype B strains may also be *transient* in the gut. But this would then indicate alternative functions for the SpaCBA pili, such as binding to other mucosa. This brings us to raise one question: is *L. rhamnosus* only specific to the human host or are there any other potential animal reservoirs? Addressing the host specificity of *L. rhamnosus* would potentially lead to identifying novel host-specific strains and remarkable adaptation patterns as reported in the species *L. reuteri* [45].

The clinical isolates constitute a very eclectic pool of strains, whose genotype and phenotype do not reflect adaptation patterns of their source of isolation, *i.e.* blood or pus, but rather of their original ecological niche. Thirty-three clinical strains could be assigned to one distinct geno-phenotype, *i.e.* 10 isolates with geno-phenotype A, 9 isolates with geno-phenotype B and 14 isolates with geno-phenotype B^ΔspaCBA^. The geno-phenotypes B and B^ΔspaCBA^ differ by the presence or lack of the genomic island containing the *spaCBA* pili gene cluster, which is located in an unstable genomic region [64]. A number of other strains (*n* = 17) were not be assigned any geno-phenotype, as they possess transitory geno-phenotypes or may have atypical history. It is noteworthy, that some of the clinical isolates have similar gene content to *L. rhamnosus* GG. However, differences in phenotypes clearly show that these strains are not identical to GG. This indicates that they may have additional genes and/or nucleotide variations in their respective genomes and share close ancestor to *L. rhamnosus* GG. This is in line with a previous study that showed that the widespread and increasing use of probiotic strain *L. rhamnosus* GG was not associated with the augmentation of *Lactobacillus* bacteremia [37].

To conclude, this work represents the first extensive genomic and functional analysis of the species *L. rhamnosus* and provides further insights into the genetics and lifestyle of this species. The data and model presented here may serve as a basis to understand the ecology of novel *L. rhamnosus* isolates, to identify novel probiotic candidates and also to examine the functional properties of current commercial *L. rhamnosus* strains.

## Materials and Methods

### *L. rhamnosus* isolate collection, DNA isolation and molecular typing

All 100 *Lactobacillus rhamnosus* strains used in this study were obtained from various institutions, universities and hospitals (Table S1). Well-characterized, *L. rhamnosus* GG was used as reference strain throughout the study [15], [19], [43]. Strains VIFIT, IDOF, AKRO, CORO and NEO were isolated from probiotic-marketed products (Table S1), whereas a number of strains were made available from strain collections or institutions. All isolates were routinely propagated in anaerobic conditions at 37°C in MRS medium (Difco BD, NJ, USA). Chromosomal DNA from each isolate was extracted using Wizard Genomic DNA Purification Kit (Promega, WI, USA) following the manufacturer's instructions. Initial bacterial identification at the species level was performed by amplification of *tuf* gene as described by Ventura *et al.* [72], [73] using standard PCR amplification conditions and multiplex PCR amplification (data not shown).

### Fermentative profile

Sugar metabolism and other catabolic properties of the *L. rhamnosus* strains were investigated using API CH 50 kit (bioMerieux, Marcy L'Etoile, France). All strains were grown until logarithmic phase and then inoculated in API galleries following the manufacturer's instructions. API galleries were further incubated at 37°C in anaerobic conditions for 48 h prior to colorimetric analysis.

### Genome SOLiD sequencing and bioinformatic sequence analysis

Genomes of all *L. rhamnosus* isolates were sequenced on a SOLiD sequencer platform (Life Technologies) at the Institute of Biotechnology (Helsinki, Finland). Sequence alignments and consensus sequences were generated by mapping color-space reads to the *L. rhamnosus* GG reference genome, using the SOLiD BioScope software (Life Technologies) and the SAM tools [74]. In order to transfer annotation from a reference genome (*L. rhamnosus* GG) to each un-annotated mapped genome, sequences were compared with ‘nucmer’ to identify regions that share synteny [75]. Those regions were extracted as base range in the mapped genome and in the reference genome (*L. rhamnosus* GG). In-house custom-made scripts were then used to transfer annotation. Synteny blocks had a nucleotide sequence identity more than or equal to 40%. For each query genome, a set of shared *L. rhamnosus* GG orthologous genes was obtained and further analyzed. Similarly, for a number of strains, we mapped the SOLiD reads onto the LC705 genome sequence and obtained an additional set of shared *L. rhamnosus* LC705 orthologous genes. The *L. rhamnosus* GG genome was assigned to COGs using Reverse Position Specific blast and Conserved Domain Database from NCBI. Mapped genome sequences are available upon request.

### Human mucus binding assay

Human intestinal mucus was kindly collected and provided by S. Vesterlund (University of Turku, Finland) and H. Huhtinen (Turku University Central Hospital, Turku, Finland) as previously described [27], [76]. *L. rhamnosus* strains were propagated and radiolabeled overnight in MRS broth supplemented with 10 µl.ml^−1^ [5′-^3^H] thymidine (16.7 Ci .mmol^−1^). MaxiSorp microtiter plates (Nunc, Denmark) were coated with 100 µL of human mucus solution prepared in PBS at a final concentration of 0.5 mg/mL and further incubated overnight at 4°C. The wells were then washed with PBS to remove unbound mucus and 100 µL of ^3^H-radiolabeled bacterial suspensions at optical density (OD_600_) 0.25±0.01 were added to the wells. The microtiter plate was further incubated at 37°C for 1 h and then wells were washed with PBS in order to remove unbound bacteria. Bacteria adhering to mucus were incubated at 60°C for 1 h in 1% SDS-0.1 M NaOH solution and the radioactivity level of lyzed bacterial suspensions was measured by liquid scintillation counting in a Wallac 1414 liquid scintillation counter (PerkinElmer). The percentage ratio between radioactivity values of lysed *L. rhamnosus* suspension (mucus-bound fraction) and *L. rhamnosus* suspension (unbound fraction) reflects the adhesion ability to human intestinal mucus. For each isolate the experiment was performed in quadruplicate.

### Antiserum-mediated human mucus binding assay

Human mucus binding assay was performed for *L. rhamnosus* isolates in the presence of polyclonal SpaC antibody as described above. ^3^H radio radiolabeled bacteria were co-incubated with the immobilized mucus in the presence of a 1∶100 dilution of anti-SpaC serum.

### Immunoblotting analysis of cell wall proteins

For each isolate, bacterial suspension adjusted to an optical density (OD_600_) of 1.0 was used to extract cell wall-associated proteins. Cell pellets were washed once with PBS and disrupted mechanically by bead-beating using sterile quartz beads (Merck KGaA, Germany). Cell wall material was resuspended in 500 µL of PBS and further pelleted by centrifugation at high speed for 30 min. Next, the samples were digested for 3 h at 37°C in a 50 µL enzymatic mixture containing 50 mM Tris-HCl, 5 mM MgCl_2_, 5 mM CaCl_2_, 10 mg/mL lysozyme and 150 U/mL mutanolysin. Samples were mixed with 12.5 µL of 4× Laemmli loading buffer (BioRad, CA, USA) and heated at 99°C for 10 min. Cell wall proteins were resolved on 10% acrylamide gel and electroblotted onto 0.2 µm nitrocellulose membrane (BioRad, CA, USA). Polyclonal rabbit SpaA antiserum (1∶10,000) and peroxidase-conjugated goat anti-rabbit IgG (Jackson ImmunoResearch, USA) (1∶10,000) were respectively used as a primary and secondary antibody in 2% (w/v) ECL Prime Blocking Reagent (GE Healthcare Life Science, UK). Membranes were blocked with 2% (w/v) ECL Prime Blocking Reagent, and washed with 0.1% Tween 20 – PBS solution in-between incubations. Membranes were analyzed using Amersham ECL Prime Western Blotting Detection Reagent (GE Healthcare Life Science, UK).

### Host signallings

HEK-Blue hTLR2/4/5 cell lines (Invivogen, CA, USA) were used in this assay. All cell lines were grown and subcultured up to 70–80% of confluency using as a maintenance medium Dulbecco's Modified Eagle Medium (DMEM) supplemented with 4.5 g/L D-glucose, 50 U/mL penicillin, 50 µg/mL streptomycin, 100 µg/mL Normocin, 2 mM L-glutamine, and 10% (v/v) of heat-inactivated fetal bovine serum. For each cell line, the immune response assay was carried out by splitting HEK-Blue cells in flat-bottom 96-well plates and stimulating them by addition of 20 µl bacterial suspensions adjusted to OD_600_ 1, 1∶10, 1∶100. The 96-well plates were incubated for 20–24 h at 37°C in a 5% CO_2_ incubator. Receptor ligands as Pam3CSK4 (100 ng/mL for hTLR2), LPS-EB (100 ng/mL for hTLR4) and RecFLA-ST (10 ng/mL for hTLR5) were used as positive control while maintenance medium without any selective antibiotics was used as negative control. SEAP secretion was detected by measuring the OD_600_ at 15 min, 1 h, 2 h, and 3 h after addition of 180 µL of QUANTI-Blue (Invivogen, CA, USA) to 20 µL of induced HEK-Blue hTLR2/4/5 supernatant. All cell lines were stimulated in triplicate for each isolate.

### TEM sample preparation

Selected *L. rhamnosus* isolates were analyzed by transmission electron microscopy (TEM) as previously described by Reunanen *et al.* [20]. Briefly, 20 µL of overnight bacterial cultures were added to Formvar-carbon-coated copper grids for 30 min at room temperature. Grids were then washed three times with 0.02 M glycine solution and further incubated for 15 min in a blocking solution containing 1% (w/v) of bovin serum albumin (BSA). Next, a 1∶100 dilution of SpaA antibody was prepared in 1% (w/v) BSA solution and added to the grids for 1 h, washed with 0.1% (w/v) BSA and incubated for 20 min with protein A conjugated to 10 nm gold particles. Grids were washed several times in PBS, fixed for 5 min using 1% glutaraldehyde, washed again with deionized water and stained with a solution containing 1.8% methycellulose and 0.4% uranyl acetate. Grids were visualized using JEOL JEM-1400 transmission electron microscope (JEOL Ltd., Japan).

### Bile resistance assay

*L. rhamnosus* strains were cultured in MRS broth at 37°C in anaerobic conditions. The OD_600_ of the bacterial culture suspensions were equalized to 1.5 and 3 µl of cell suspensions were spotted onto MRS agar plates containing 0.5% (w/v) Ox gall (Sigma, MO, USA). Plates were incubated anaerobically at 37°C for two days and visually examined.

### Antagonistic assay

*L. rhamnosus* strains were grown until stationary phase as described above. Next, the cell suspensions were thoroughly homogenized and the OD_600_ was equalized. Cell mixtures were then centrifuged for 20 min at 650×g at +5°C and the supernatants were pH-adjusted at 5.0 and 6.20 by addition of NaOH and HCl solutions, filtered (0.22 µm filter) and stored at −20°C for further analysis. Antagonistic assays were performed in microtiter well plates as previously described [77]. *E. coli* O157:H7 (ATCC 43894), *L. monocytogenes* R14-2-2 and *Y. enterocolitica* R5-9-1 were incubated for 15 h at 37°C in the presence of 30 µl of *L. rhamnosus* pH-adjusted supernatant. As positive controls, 30 µl of sterile MRS broth at pH 6.20 or pH 5.0 was added the dedicated medium (TSB or LB) inoculated with one of the pathogenic strains. As a negative control, 300 µl of medium (TSB or LB) was used. The OD_600_ values were measured in an automatic reader (Bioscreen C, Oy Growth Curves Ab Ltd, Finland) every 30 min. The bacterial growth was quantified using growth curves and the area under curve (AUC) values, automatically processed by the BioLink software (Oy Growth Curves Ab). Inhibition was expressed as an area reduction percentage (ARP) compared to control samples grown without the addition of supernatant.

## Supporting Information

Figure S1 COG distribution in *L. rhamnosus* core genome, *L. rhamnosus* GG genome and GG-specific gene subset.
(TIF)

Figure S2 Overview of the 17 variable regions reported in 100 *L. rhamnosus* strains. The frequency of gene loss was calculated for each *L. rhamnosus* GG gene and plotted on the X-axis that represents GG chromosome. Each region is numbered as described in Table 2. In addition, other regions labelled as follows: i for IS elements, ii for conserved proteins, iii for metabolism-associated genes.
(TIF)

Figure S3 Adhesion of *L. rhamnosus* strains to human mucus in the presence of SpaC anti-serum. Radiolabeled (^3^H) cells of 23 different *L. rhamnosus* isolates were tested in the presence or the absence of serum directed against SpaC pilin subunit. The experiment was performed in triplicates.
(TIF)

Figure S4 Examples of *L. rhamnosus* strains analyzed by transmission electron microscopy. Ten *L. rhamnosus* strains were labelled with anti-SpaA gold particles and observed by electron microscopy. Arrows indicates pili structures. Legend: A for GG; B for H1249; C for H1242; D for H1031; E for H1094; F for H1180; G for H1101; H for H1102; I for H1225; J for H1129.
(TIF)

Figure S5 TLR-2 response of HEK-Blue cell line to *L. rhamnosus* strains. HEK-Blue hTLR2 cells were co-incubated in HEK-Blue medium in the presence of *L. rhamnosus* strains. After 1 h, NF-κB-induced SEAP activity was quantified by spectrophotometry.
(TIF)

Figure S6 Anti-microbial activity of *L. rhamnosus* strains against *E. coli*, *Yersina enterocolica* and *Listeria monocytogenes*. Ninety-two *L. rhamnosus* strains were tested for potential anti-microbial activity as described in the Materials and Methods section. The filtrates used in the experiment were adjusted at two different pH: 5.0 and 6.2. Colour legend for the heat map: green for significant anti-microbial activity, black for no activity and red for inverse effect. Colour legend for the *L. rhamnosus* strains: green for dairy isolates and red for human isolates.
(TIF)

Table S1 Strains have been obtained or isolated from various institutions and labelled as follows: FIN-U for Department of Veterinary Biosciences, Helsinki University, Finland; FIN-V for Valio Culture Collection Ltd., Helsinki, Finland; ITA-C for Department of Microbiology and Food Technology, University of Catania, Italy; ITA-F for Department of Bio-Medical Sciences, Microbiology section, University of Catania, Italy; ITA-P for Department of Genetics, Biology of Microorganisms, Anthropology, Evolution, University of Parma, Parma, Italy; IRL for TEAGASC & Alimentary Pharmabiotic Centre, UCC, Cork, Ireland and NL-Y for Yoba for Life Foundation, Amsterdam, The Netherlands. Strains obtained from Valio Culture Collection Ltd. were initially isolated and collected by the HUSLAB (Helsinki University Central Hospital Laboratory, Helsinki) and other clinical laboratories around Finland, some of them having been described in previous epidemiological studies [37], [80]. Legend: CC for colour code; strains were coloured according their ecological niches or isolation sources.
(DOCX)

Table S2 List of genes present in GG and missing in at least one strain. The core genome of the *L. rhamnosus* species can be deduced from the present gene list.
(DOCX)

Table S3 Comparative genomic data of 100 *L. rhamnosus* strains. Legend: 1 for gene present in that particular strain and 0 for divergent/missing gene.
(XLSX)

Table S4 BLAST analysis of the spacers present in *L. rhamnosus* GG CRISPR locus. Each spacer was blasted using NCBI BlastN using the default parameters with the following modifications: word size 7, expected threshold 0.1, optimized for ‘somewhat similar’.
(DOCX)
