# Supplementary figures and images for: Comparative Genomic and Functional Analysis of 100 Lactobacillus rhamnosus Strains and Their Comparison with Strain GG
Source: PLoS Genet. 2013 Aug 15;9(8):e1003683. doi: 10.1371/journal.pgen.1003683 (PMC3744422; doi:10.1371/journal.pgen.1003683)

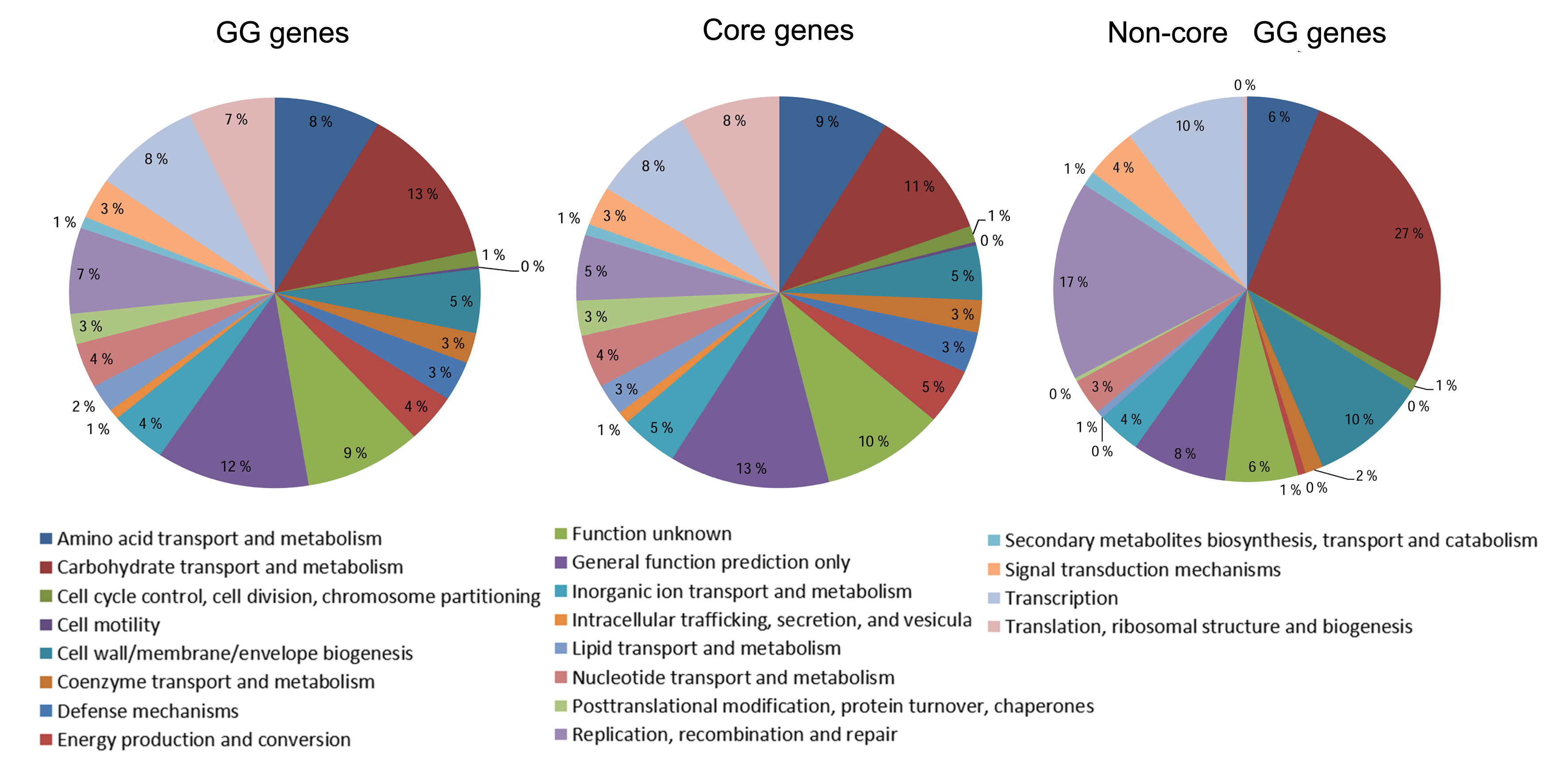

Supplement: Figure S1 — COG distribution in L. rhamnosus core genome, L. rhamnosus GG genome and GG-specific gene subset. (TIF) [file pgen.1003683.s001.tif]

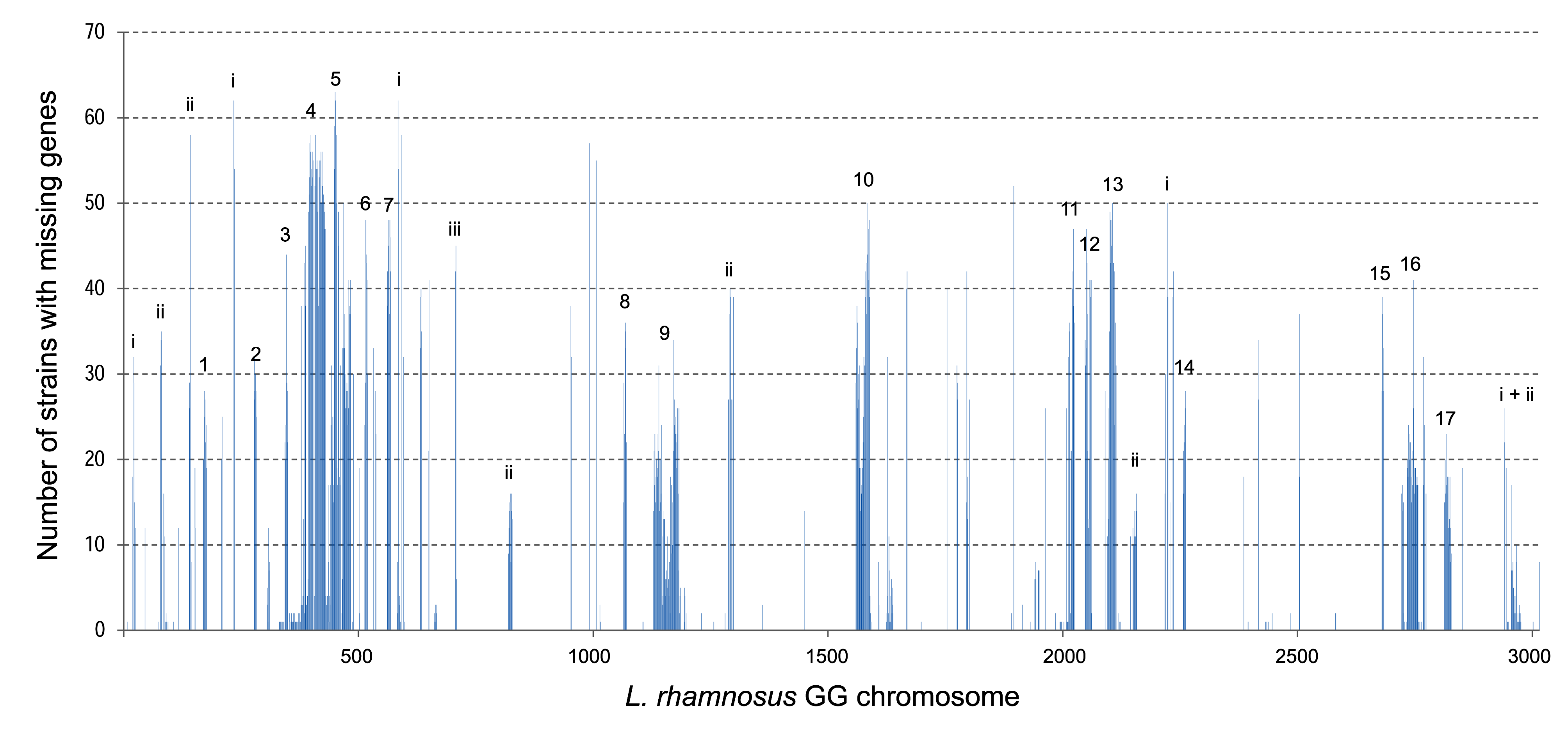

Supplement: Figure S2 — Overview of the 17 variable regions reported in 100 L. rhamnosus strains. The frequency of gene loss was calculated for each L. rhamnosus GG gene and plotted on the X-axis that represents GG chromosome. Each region is numbered as described in Table 2. In addition, other regions labelled as follows: i for IS elements, ii for conserved proteins, iii for metabolism-associated genes. (TIF) [file pgen.1003683.s002.tif]

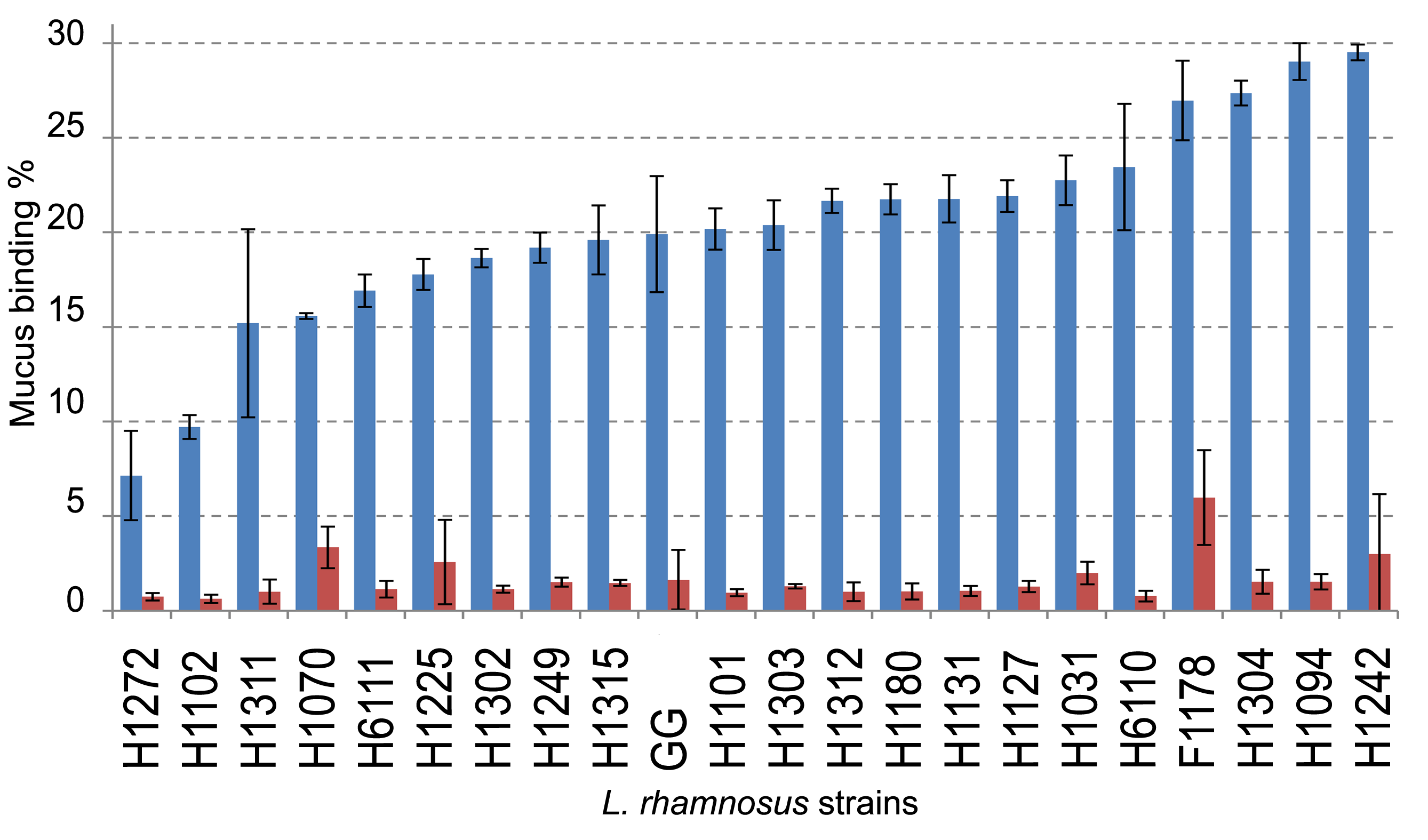

Supplement: Figure S3 — Adhesion of L. rhamnosus strains to human mucus in the presence of SpaC anti-serum. Radiolabeled (3H) cells of 23 different L. rhamnosus isolates were tested in the presence or the absence of serum directed against SpaC pilin subunit. The experiment was performed in triplicates. (TIF) [file pgen.1003683.s003.tif]

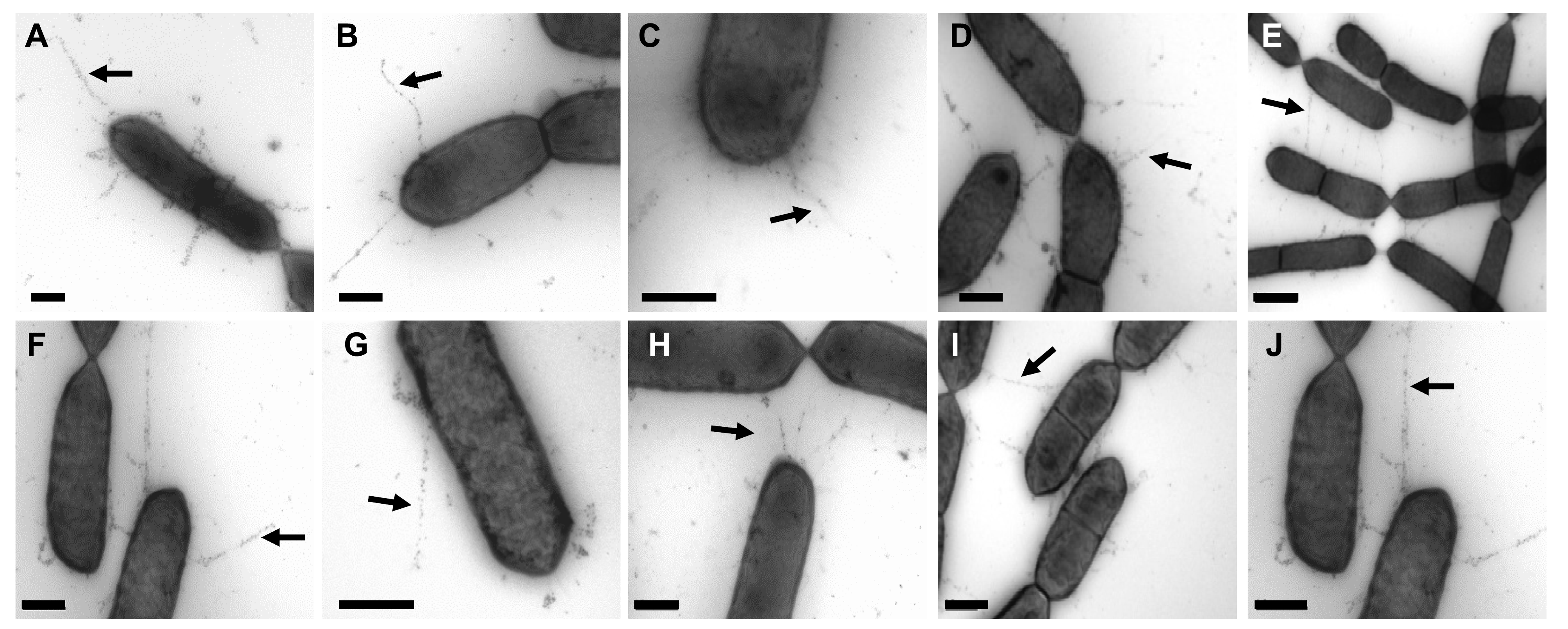

Supplement: Figure S4 — Examples of L. rhamnosus strains analyzed by transmission electron microscopy. Ten L. rhamnosus strains were labelled with anti-SpaA gold particles and observed by electron microscopy. Arrows indicates pili structures. Legend: A for GG; B for H1249; C for H1242; D for H1031; E for H1094; F for H1180; G for H1101; H for H1102; I for H1225; J for H1129. (TIF) [file pgen.1003683.s004.tif]

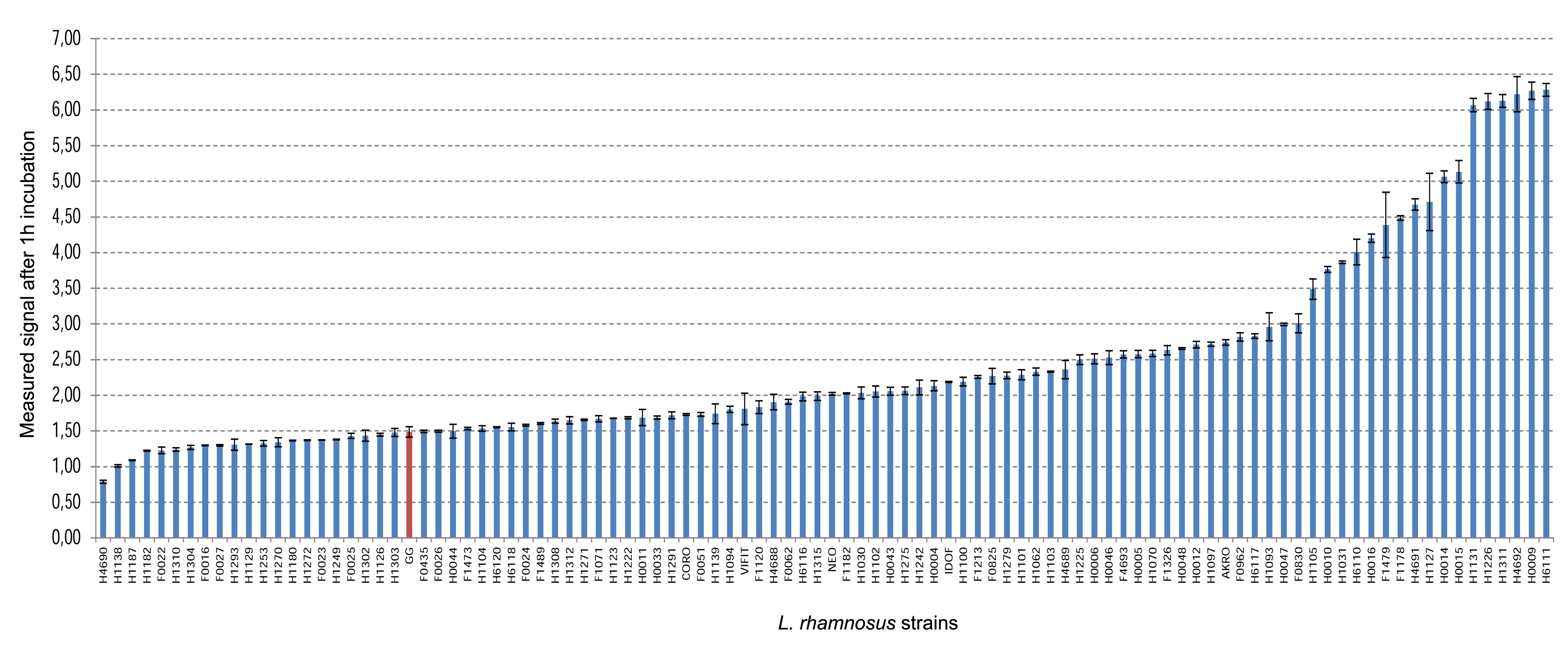

Supplement: Figure S5 — TLR-2 response of HEK-Blue cell line to L. rhamnosus strains. HEK-Blue hTLR2 cells were co-incubated in HEK-Blue medium in the presence of L. rhamnosus strains. After 1 h, NF-κB-induced SEAP activity was quantified by spectrophotometry. (TIF) [file pgen.1003683.s005.tif]

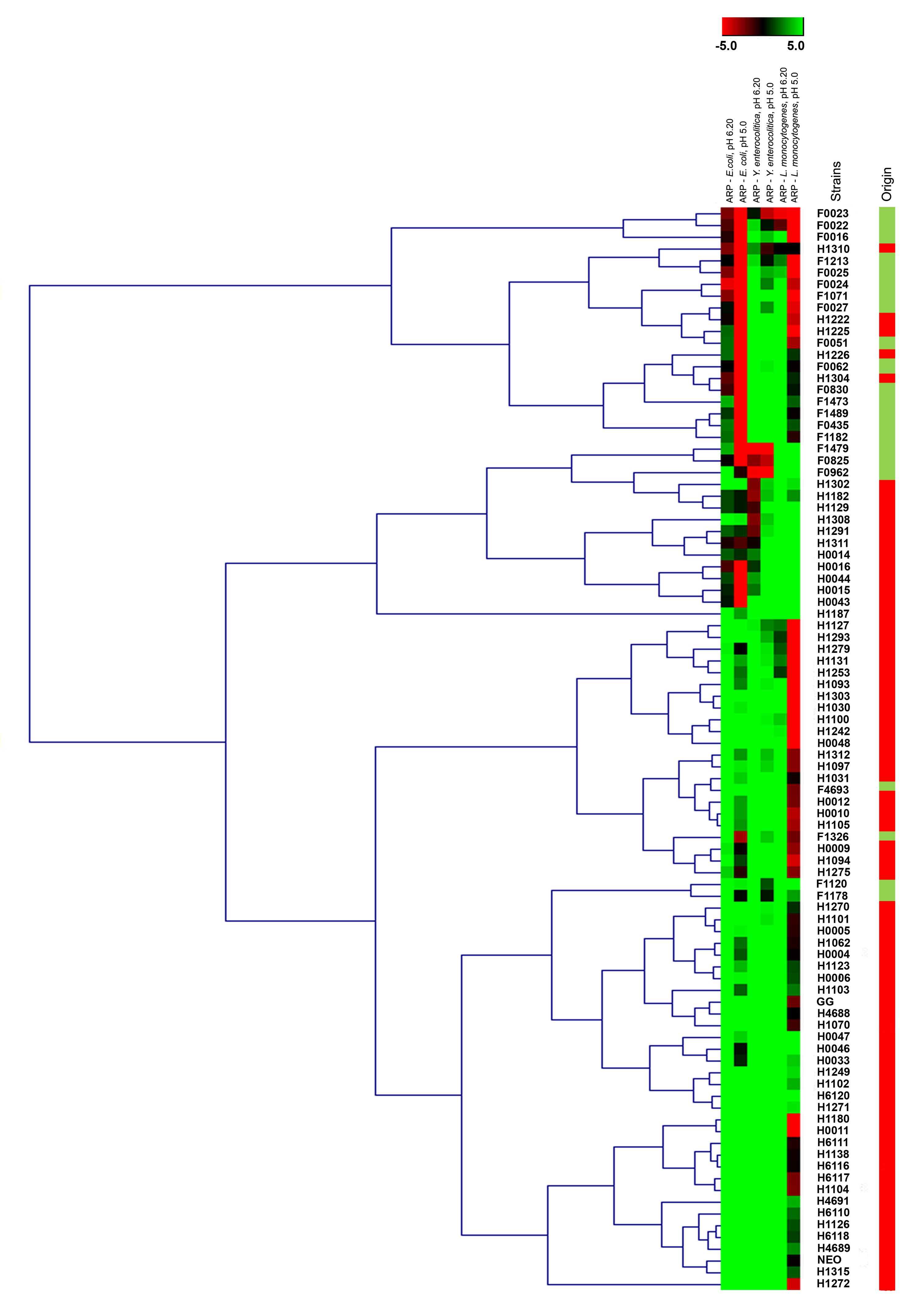

Supplement: Figure S6 — Anti-microbial activity of L. rhamnosus strains against E. coli, Yersina enterocolica and Listeria monocytogenes. Ninety-two L. rhamnosus strains were tested for potential anti-microbial activity as described in the Materials and Methods section. The filtrates used in the experiment were adjusted at two different pH: 5.0 and 6.2. Colour legend for the heat map: green for significant anti-microbial activity, black for no activity and red for inverse effect. Colour legend for the L. rhamnosus strains: green for dairy isolates and red for human isolates. (TIF) [file pgen.1003683.s006.tif]
