# Supplementary material for: Comparative Genomic and Functional Analysis of 100 Lactobacillus rhamnosus Strains and Their Comparison with Strain GG
Source: PLoS Genet. 2013 Aug 15;9(8):e1003683. doi: 10.1371/journal.pgen.1003683 (PMC3744422; doi:10.1371/journal.pgen.1003683)
Supplement: Table S1 — Strains have been obtained or isolated from various institutions and labelled as follows: FIN-U for Department of Veterinary Biosciences, Helsinki University, Finland; FIN-V for Valio Culture Collection Ltd., Helsinki, Finland; ITA-C for Department of Microbiology and Food Technology, University of Catania, Italy; ITA-F for Department of Bio-Medical Sciences, Microbiology section, University of Catania, Italy; ITA-P for Department of Genetics, Biology of Microorganisms, Anthropology, Evolution, University of Parma, Parma, Italy; IRL for TEAGASC & Alimentary Pharmabiotic Centre, UCC, Cork, Ireland and NL-Y for Yoba for Life Foundation, Amsterdam, The Netherlands. Strains obtained from Valio Culture Collection Ltd. were initially isolated and collected by the HUSLAB (Helsinki University Central Hospital Laboratory, Helsinki) and other clinical laboratories around Finland, some of them having been described in previous epidemiological studies [37], [80]. Legend: CC for colour code; strains were coloured according their ecological niches or isolation sources. (DOCX) [file pgen.1003683.s007.docx]

| # | Strain Name | Names used in the study | Source | Institute | **CC** |
| --- | --- | --- | --- | --- | --- |
| 1 | AK-RO-01 | AKRO | Yoba yogurt | NL-Y |  |
| 2 | CO-RO-01 | CORO | Yoba yogurt | NL-Y |  |
| 3 | D16 | F0016 | Pecorino cheese (60 days) | ITA-C |  |
| 4 | D22 | F0022 | Pecorino cheese (60 days) | ITA-C |  |
| 5 | D24 | F0023 | Pecorino cheese (60 days) | ITA-C |  |
| 6 | E24 | F0024 | Pecorino cheese (60 days) | ITA-C |  |
| 7 | H25 | F0025 | Pecorino cheese (120 days) | ITA-C |  |
| 8 | E26 | F0026 | Pecorino cheese (60 days) | ITA-C |  |
| 9 | D26 | F0027 | Pecorino cheese (60 days) | ITA-C |  |
| 10 | H51 | F0051 | Pecorino cheese (120 days) | ITA-C |  |
| 11 | E62 | F0062 | Pecorino cheese (60 days) | ITA-C |  |
| 12 | 435 | F0435 | Pannerone cheese | ITA-P |  |
| 13 | 825 | F0825 | Parmigiano Reggiano curd | ITA-P |  |
| 14 | 830 | F0830 | Parmigiano Reggiano curd | ITA-P |  |
| 15 | 962 | F0962 | Parmigiano Reggiano cheese (90 days) | ITA-P |  |
| 16 | 1071 | F1071 | Parmigiano Reggiano cheese (8 months) | ITA-P |  |
| 17 | 1120 | F1120 | Parmigiano Reggiano cheese (10 months) | ITA-P |  |
| 18 | 1178 | F1178 | Parmigiano Reggiano cheese (12 months) | ITA-P |  |
| 19 | 1182 | F1182 | Parmigiano Reggiano cheese (12 months) | ITA-P |  |
| 20 | 1213 | F1213 | Parmigiano Reggiano cheese (12 months) | ITA-P |  |
| 21 | 1326 | F1326 | Parmigiano Reggiano cheese (16 months) | ITA-P |  |
| 22 | 1473 | F1473 | Parmigiano Reggiano cheese (20 months) | ITA-P |  |
| 23 | 1479 | F1479 | Parmigiano Reggiano cheese (20 months) | ITA-P |  |
| 24 | 1489 | F1489 | Parmigiano Reggiano cheese (20 months) | ITA-P |  |
| 25 | APC 4693 | F4693 | hard cheese, LMG 12166 | IRL |  |
| 26 | Lac 4 | H0004 | vaginal cavity | ITA-F |  |
| 27 | Lac 5 | H0005 | vaginal cavity | ITA-F |  |
| 28 | Lac 6 | H0006 | vaginal cavity | ITA-F |  |
| 29 | Lac 9 | H0009 | vaginal cavity | ITA-F |  |
| 30 | Lac 10 | H0010 | vaginal cavity | ITA-F |  |
| 31 | Lac 11 | H0011 | vaginal cavity | ITA-F |  |
| 32 | Lac 12 | H0012 | vaginal cavity | ITA-F |  |
| 33 | E14Cork | H0014 | intestinal tract | IRL |  |
| 34 | E16a | H0015 | intestinal tract | IRL |  |
| 35 | E16b | H0016 | intestinal tract | IRL |  |
| 36 | Lac 33 | H0033 | oral cavity | ITA-F |  |
| 37 | E43 Cork | H0043 | intestinal tract | IRL |  |
| 38 | E44 Cork | H0044 | intestinal tract | IRL |  |
| 39 | Lac 46 | H0046 | oral cavity | ITA-F |  |
| 40 | E47 Cork | H0047 | clinical isolate | IRL |  |
| 41 | Lac 48 | H0048 | oral cavity | ITA-F |  |
| 42 | 1030 (T 4813) | H1030 | blood | FIN-V |  |
| 43 | 1031 (T 4846) | H1031 | blood | FIN-V |  |
| 44 | 1062 (T 24029) | H1062 | blood | FIN-V |  |
| 45 | 1070 (T 25865) | H1070 | blood | FIN-V |  |
| 46 | 1093 (T 23808) | H1093 | blood | FIN-V |  |
| 47 | 1094 (T 70980) | H1094 | blood | FIN-V |  |
| 48 | 1097 (T 70977) | H1097 | blood | FIN-V |  |
| 49 | 1100 (T 71004) | H1100 | blood | FIN-V |  |
| 50 | 1101 (T 71005) | H1101 | blood | FIN-V |  |
| 51 | 1102 (T 71006) | H1102 | blood | FIN-V |  |
| 52 | 1103 (T 71007) | H1103 | blood | FIN-V |  |
| 53 | 1104 (T 71009) | H1104 | blood | FIN-V |  |
| 54 | 1105 (T 71034) | H1105 | abscess | FIN-V |  |
| 55 | 1123 (T 71273) | H1123 | clinical isolate | FIN-V |  |
| 56 | 1126 (T 71311) | H1126 | clinical isolate | FIN-V |  |
| 57 | 1127 (T 71326) | H1127 | clinical isolate | FIN-V |  |
| 58 | 1129 (T 71330) | H1129 | clinical isolate | FIN-V |  |
| 59 | 1131 (ME 8296) | H1131 | pus | FIN-V |  |
| 60 | 1138 (T 32154) | H1138 | clinical isolate | FIN-V |  |
| 61 | 1139 (T 71353) | H1139 | blood | FIN-V |  |
| 62 | 1180 (T 33620) | H1180 | blood | FIN-V |  |
| 63 | 1182 (T 71755) | H1182 | clinical isolate | FIN-V |  |
| 64 | 1187 (T 33651) | H1187 | blood | FIN-V |  |
| 65 | 1222 (T 15756) | H1222 | blood | FIN-V |  |
| 66 | 1225 (T 21162) | H1225 | blood | FIN-V |  |
| 67 | 1226 (T 19557) | H1226 | blood | FIN-V |  |
| 68 | 1242 (T 36186) | H1242 | blood | FIN-V |  |
| 69 | 1249 (T 38522) | H1249 | blood | FIN-V |  |
| 70 | 1253 (T 72663) | H1253 | blood | FIN-V |  |
| 71 | 1270 (T 73573) | H1270 | clinical isolate | FIN-V |  |
| 72 | 1271 (T 73572) | H1271 | clinical isolate | FIN-V |  |
| 73 | 1272 (T 38983) | H1272 | blood | FIN-V |  |
| 74 | 1275 (T 39685) | H1275 | blood | FIN-V |  |
| 75 | 1279 (T 41773) | H1279 | blood | FIN-V |  |
| 76 | 1291 (T 74230) | H1291 | blood | FIN-V |  |
| 77 | 1293 (T 74232) | H1293 | blood | FIN-V |  |
| 78 | 1302 (T 74293) | H1302 | clinical isolate | FIN-V |  |
| 79 | 1303 (T 74294) | H1303 | clinical isolate | FIN-V |  |
| 80 | 1304 (T 74236) | H1304 | clinical isolate | FIN-V |  |
| 81 | 1308 (T 42258) | H1308 | blood | FIN-V |  |
| 82 | 1310 (T 43966) | H1310 | blood | FIN-V |  |
| 83 | 1311 (T 42376) | H1311 | blood | FIN-V |  |
| 84 | 1312 (T 74518) | H1312 | blood | FIN-V |  |
| 85 | 1315 (T 74493) | H1315 | clinical isolate | FIN-V |  |
| 86 | APC 4688 (CCUG23641) | H4688 | blood | IRL |  |
| 87 | APC 4689 | H4689 | hip punction | IRL |  |
| 88 | APC 4690 | H4690 | LMG 6400Ta-type strain | IRL |  |
| 89 | APC 4691 | H4691 | LMG 6400Tb-type strain | IRL |  |
| 90 | APC 4692 (LMG 8153) | H4692 | healthy adult female urethra | IRL |  |
| 91 | APC 6110 | H6110 | infant isolate | IRL |  |
| 92 | APC 6111 | H6111 | infant isolate | IRL |  |
| 93 | APC 6116 | H6116 | infant isolate | IRL |  |
| 94 | APC 6117 | H6117 | infant isolate | IRL |  |
| 95 | APC 6118 | H6118 | infant isolate | IRL |  |
| 96 | APC 6120 | H6120 | infant isolate | IRL |  |
| 97 | Idoform LGG | IDOF | probiotic-marketed product | FIN-U |  |
| 98 | *L. rhamnosus* LGG | GG | intestinal tract, used in dairy products | FIN-U |  |
| 99 | Neo-Imunele | NEO | probiotic-marketed yogurt | FIN-U |  |
| 100 | Vifit LGG | VIFIT | probiotic-marketed drinkable yogurt | FIN-U |  |
