# Supplementary material for: Comparative Genomic and Functional Analysis of 100 Lactobacillus rhamnosus Strains and Their Comparison with Strain GG
Source: PLoS Genet. 2013 Aug 15;9(8):e1003683. doi: 10.1371/journal.pgen.1003683 (PMC3744422; doi:10.1371/journal.pgen.1003683)
Supplement: Table S2 — List of genes present in GG and missing in at least one strain. The core genome of the L. rhamnosus species can be deduced from the present gene list. (DOCX) [file pgen.1003683.s008.docx]

| **Gene Name** | **Symbol** | **Predicted Gene Product** |
| --- | --- | --- |
| LGG_00009 | LGG_00009 | Putative protein without homology |
| LGG_00020 | is1 | Transposase, IS30 family protein |
| LGG_00022 | is2 | Transposase, IS5 family protein |
| LGG_00023 | LGG_00023 | Metal-dependent membrane protease |
| LGG_00024 | LGG_00024 | Putative protein without homology |
| LGG_00026 | is3 | Transposase, IS5 family protein |
| LGG_00046 | LGG_00046 | Putative protein without homology |
| LGG_00074 | LGG_00074 | Conserved protein |
| LGG_00079 | LGG_00079 | Conserved protein |
| LGG_00080 | zntR | Transcriptional regulator, MerR family |
| LGG_00081 | LGG_00081 | Conserved protein |
| LGG_00086 | LGG_00086 | Transcriptional regulator, TetR family |
| LGG_00087 | LGG_00087 | Conserved protein |
| LGG_00090 | LGG_00090 | PTS system, IICB component |
| LGG_00091 | LGG_00091 | Putative protein without homology |
| LGG_00092 | frvA | PTS system, IIA component |
| LGG_00095 | bglB | Beta-glucosidase (GH1) |
| LGG_00107 | rmlC | dTDP-4-dehydrorhamnose 3,5-epimerase |
| LGG_00117 | LGG_00117 | Transcriptional regulator |
| LGG_00140 | uvrB | Conserved protein |
| LGG_00141 | LGG_00141 | Putative protein without homology |
| LGG_00143 | LGG_00143 | Conserved protein |
| LGG_00144 | is4 | Transposase |
| LGG_00152 | is5 | Transposase |
| LGG_00153 | is6 | Transposase, IS4 family protein |
| LGG_00170 | LGG_00170 | Putative protein without homology |
| LGG_00171 | LGG_00171 | Conserved protein |
| LGG_00172 | tauB | ABC transporter, taurine transporter ATP-b |
| LGG_00173 | tauA | ABC transporter, aliphatic sulfonates fami |
| LGG_00174 | tauC | Taurine transport system permease protein |
| LGG_00175 | naoX | Pyridine nucleotide-disulphide oxidoreduct |
| LGG_00176 | LGG_00176 | Conserved membrane protein |
| LGG_00177 | LGG_00177 | Transcriptional regulator, LysR family |
| LGG_00209 | LGG_00209 | ABC transporter, ATPase and permease compo |
| LGG_00210 | rrg | Transcriptional regulator, xre family |
| LGG_00235 | is7 | Transposase |
| LGG_00236 | is8 | Transposase, IS4 family protein |
| LGG_00278 | LGG_00278 | Conserved protein |
| LGG_00279 | welA | dTDP-rhamnosyl transferase rfbF |
| LGG_00280 | welB | alpha-L-Rha alpha-1,3-L-rhamnosyltransfera |
| LGG_00281 | welC | alpha-L-Rha alpha-1,3-L-rhamnosyltransfera |
| LGG_00282 | eps1 | Polysaccharide Transporter, PST family pro |
| LGG_00283 | eps2 | CpsH |
| LGG_00305 | LGG_00305 | Conserved protein |
| LGG_00306 | LGG_00306 | Conserved protein |
| LGG_03002 | 23S rRNA | 23S ribosomal RNA |
| LGG_00307 | LGG_00307 | Conserved protein |
| LGG_00308 | LGG_00308 | Lipoprotein |
| LGG_00309 | LGG_00309 | Conserved protein |
| LGG_00329 | upgB | ABC transporter, sugar transporter peripla |
| LGG_00330 | LGG_00330 | Putative protein without homology |
| LGG_00331 | manA | Mannose-6-phosphate isomerase |
| LGG_00332 | gntR | Transcriptional regulator, GntR family |
| LGG_00333 | agaS | Tagatose-6-phosphate ketose/aldose isomera |
| LGG_00336 | bgaC | Beta-galactosidase (GH35) |
| LGG_00338 | manC | PTS system, mannose-specific IIC component |
| LGG_00339 | manD | PTS system, mannose-specific IID component |
| LGG_00341 | lacC | Tagatose-6-phosphate kinase |
| LGG_00342 | srlD | Sorbitol-6-phosphate 2-dehydrogenase |
| LGG_00343 | gatC | PTS system, galactitol-specific IIC compon |
| LGG_00344 | is9 | Transposase, IS5 family protein |
| LGG_00345 | gatA | PTS system, galactitol-specific IIA compon |
| LGG_00346 | gatB | PTS system, galactitol-specific IIB compon |
| LGG_00347 | LGG_00347 | Conserved protein |
| LGG_00351 | patB | Aminotransferase |
| LGG_00352 | ypdF | Aminopeptidase YpdF |
| LGG_00354 | celA | PTS system, lactose/cellobiose-specific II |
| LGG_00355 | chbA | PTS system, lactose/cellobiose-specific II |
| LGG_00356 | ypdE | Aminopeptidase |
| LGG_00357 | LGG_00357 | Transcription antiterminator |
| LGG_00358 | pepT | Peptidase T |
| LGG_00359 | oppA | ABC transporter, oligopeptide-binding prot |
| LGG_00360 | yhbS | Acetyltransferase |
| LGG_00361 | LGG_00361 | Conserved protein |
| LGG_00363 | LGG_00363 | ABC transporter, cobalt transporter permea |
| LGG_00364 | cbiO | ABC transporter, cobalt transporter ATP-bi |
| LGG_00365 | cbiQ | ABC transporter, cobalt transporter permea |
| LGG_00366 | tenA | Transcriptional activator TenA |
| LGG_00367 | thiW | Hydroxyethylthiazole permease |
| LGG_00369 | thiE | Thiamine-phosphate pyrophosphorylase |
| LGG_00370 | thiD | Phosphomethylpyrimidine kinase |
| LGG_00372 | rbsR | Transcriptional regulator, LacI family |
| LGG_00373 | rbsK | Ribokinase |
| LGG_00374 | LGG_00374 | Transcriptional regulator |
| LGG_00375 | LGG_00375 | Putative protein without homology |
| LGG_00376 | is10 | Transposase, IS5 family protein |
| LGG_00377 | LGG_00377 | Putative protein without homology |
| LGG_00378 | LGG_00378 | Putative protein without homology |
| LGG_00379 | LGG_00379 | Putative protein without homology |
| LGG_00380 | LGG_00380 | Conserved protein |
| LGG_00381 | LGG_00381 | Conserved protein |
| LGG_00382 | pbp | Penicillin-binding protein 3 |
| LGG_00383 | is11 | Transposase, IS5 family protein |
| LGG_00384 | LGG_00384 | Putative protein without homology |
| LGG_00385 | LGG_00385 | Putative protein without homology |
| LGG_00386 | LGG_00386 | Transporter, major facilitator superfamily |
| LGG_00387 | slyA | Transcriptional regulator, MarR family |
| LGG_00388 | LGG_00388 | Conserved protein |
| LGG_00389 | LGG_00389 | Putative protein without homology |
| LGG_00390 | aroE | Conserved protein |
| LGG_00391 | LGG_00391 | Putative protein without homology |
| LGG_00392 | LGG_00392 | Putative protein without homology |
| LGG_00393 | manR | Transcription antiterminator BglG family p |
| LGG_00394 | LGG_00394 | PTS system, IIA component |
| LGG_00395 | frwA1 | PTS system, fructose-specific IIA componen |
| LGG_00396 | frwB1 | PTS system, fructose-specific IIB componen |
| LGG_00397 | frwC1 | PTS system, fructose-specific IIC componen |
| LGG_00398 | tal | Transaldolase |
| LGG_00399 | rpe | Ribulose-phosphate 3-epimerase |
| LGG_00400 | ulaA | PTS system, lactose/cellobiose-specific II |
| LGG_00401 | ulaB | PTS system, lactose/cellobiose-specific II |
| LGG_00404 | ulaC | PTS system, ascorbate-specific IIC compone |
| LGG_00405 | tktN | Transketolase |
| LGG_00406 | tktC | Transketolase |
| LGG_00407 | scrK | Fructokinase |
| LGG_00408 | lacR | Lactose phosphotransferase system represso |
| LGG_00409 | frwA2 | PTS system, fructose-specific IIA componen |
| LGG_00410 | frwB2 | PTS system, fructose-specific IIB componen |
| LGG_00411 | frwC2 | PTS system, fructose-specific IIC componen |
| LGG_00412 | is13 | Transposase, IS30 family protein |
| LGG_00413 | fba | Fructose-bisphosphate aldolase |
| LGG_00414 | gatY | Fructose-bisphosphate aldolase |
| LGG_00415 | pts | PTS system, mannose-specific IIB component |
| LGG_00416 | pts | PTS system, mannose-specific IIA component |
| LGG_00417 | manZ | PTS system, mannose-specific IICD componen |
| LGG_00418 | tal | Transaldolase |
| LGG_00419 | LGG_00419 | Transcriptional regulator, LacI family |
| LGG_00420 | yhfZ | Transcriptional regulator, GntR family |
| LGG_00421 | LGG_00421 | Conserved protein |
| LGG_00422 | LGG_00422 | Conserved protein |
| LGG_00423 | LGG_00423 | Conserved membrane protein |
| LGG_00424 | php | Hydrolase |
| LGG_00425 | yhfS | Pyridoxal phosphate-dependent transferase |
| LGG_00426 | yhfX | Amino acid racemase |
| LGG_00427 | YHFW | Phosphopentomutase |
| LGG_00428 | ilvH | DNA-directed RNA polymerase subunit delta |
| LGG_00429 | LGG_00429 | Cobalt transport protein cbiQ |
| LGG_00430 | cbiO | ABC transporter, cobalt transporter eATP-b |
| LGG_00431 | cbiQ | Cobalt transport protein cbiQ |
| LGG_00432 | LGG_00432 | Putative protein without homology |
| LGG_00433 | menC | N-acylamino acid racemase |
| LGG_00434 | nagZ | Beta-N-acetylhexosaminidase (GH3) |
| LGG_00435 | is14 | Transposase, IS30 family protein |
| LGG_00436 | tnpR | Resolvase |
| LGG_00437 | LGG_00437 | Conserved cytosolic protein |
| LGG_00438 | LGG_00438 | Putative protein without homology |
| LGG_00439 | LGG_00439 | Putative protein without homology |
| LGG_00440 | LGG_00440 | Putative protein without homology |
| LGG_00441 | srtC1 | Sortase family protein |
| LGG_00442 | spaA | Pilus specific protein, major backbone pro |
| LGG_00443 | spaB | Pilus specific protein, minor backbone pro |
| LGG_00444 | spaC | Pilus specific protein, ancillary protein |
| LGG_00445 | is15 | Transposase, IS30 family protein |
| LGG_00446 | LGG_00446 | Conserved protein |
| LGG_00447 | LGG_00447 | Conserved protein |
| LGG_00448 | LGG_00448 | Putative protein without homology |
| LGG_00449 | LGG_00449 | UvrD/REP helicase |
| LGG_00450 | ybjD | Putative protein without homology |
| LGG_00451 | is16 | Transposase, IS5 family protein |
| LGG_00452 | is17 | Transposase, IS3/IS911 family protein |
| LGG_00453 | is18 | Transposase, IS150/IS3 family protein |
| LGG_00454 | is19 | Transposase, IS150/IS3 family protein |
| LGG_00455 | LGG_00455 | Conserved protein |
| LGG_00456 | pacL | Cation-transporting ATPase |
| LGG_00457 | is20 | Transposase |
| LGG_00458 | is21 | Transposase, IS4 family protein |
| LGG_00459 | napA | Na /H antiporter |
| LGG_00462 | is24 | Transposase |
| LGG_00463 | eriC | Chloride channel protein |
| LGG_00464 | is25 | Transposase, IS30 family protein |
| LGG_00465 | LGG_00465 | Transporter, major facilitator superfamily |
| LGG_00466 | is26 | Transposase, IS5 family protein |
| LGG_00467 | LGG_00467 | Peptidase M20 |
| LGG_00468 | LGG_00468 | Transcriptional regulator, LysR family |
| LGG_00469 | LGG_00469 | Putative protein without homology |
| LGG_00470 | ebgA | Beta-galactosidase (GH42) |
| LGG_00471 | ygjI | Amino acid permease family protein |
| LGG_00472 | lysP | Lysine-specific permease |
| LGG_00473 | araC | Transcriptional regulator, AraC family |
| LGG_00474 | LGG_00474 | Putative protein without homology |
| LGG_00475 | LGG_00475 | Transporter, major facilitator superfamily |
| LGG_00476 | abgB | Aminobenzoyl-glutamate utilization protein |
| LGG_00477 | LGG_00477 | Opine/octopine dehydrogenase |
| LGG_00478 | yecC | ABC transporter, amino acid transporter AT |
| LGG_00479 | LGG_00479 | ABC transporter, amino acid binding protei |
| LGG_00480 | LGG_00480 | ABC transporter, amino acid transporter pe |
| LGG_00481 | LGG_00481 | ABC transporter, amino acid transporter pe |
| LGG_00485 | LGG_00485 | Conserved protein |
| LGG_00487 | LGG_00487 | Putative protein without homology |
| LGG_00499 | is27 | Transposase, IS5 family protein |
| LGG_00500 | metQ | ABC transporter, metal ion transporter per |
| LGG_00511 | LGG_00511 | ABC transporter, ATP-binding protein |
| LGG_00512 | LGG_00512 | ABC transporter |
| LGG_00513 | is28 | Transposase, IS5 family protein |
| LGG_00514 | LGG_00514 | Putative protein without homology |
| LGG_00515 | LGG_00515 | Putative protein without homology |
| LGG_00516 | LGG_00516 | Transcriptional activator |
| LGG_00517 | is29 | Transposase, IS5 family protein |
| LGG_00529 | LGG_00529 | Conserved protein |
| LGG_00533 | radC | Conserved protein |
| LGG_00535 | LGG_00535 | Conserved protein |
| LGG_00559 | LGG_00559 | Transcriptional regulator, LysR family |
| LGG_00560 | LGG_00560 | Conserved protein |
| LGG_00561 | LGG_00561 | Sugar phosphate isomerase/epimerase |
| LGG_00562 | kduD | Short-chain dehydrogenase/reductase SDR |
| LGG_00563 | LGG_00563 | Putative protein without homology |
| LGG_00564 | aroE | Shikimate 5-dehydrogenase |
| LGG_00565 | yfkL | Transporter, major facilitator superfamily |
| LGG_00566 | aroE | Shikimate 5-dehydrogenase |
| LGG_00579 | LGG_00579 | Conserved extracellular protein |
| LGG_00580 | LGG_00580 | Putative protein without homology |
| LGG_00581 | is30 | Transposase |
| LGG_00582 | is31 | Transposase, IS4 family protein |
| LGG_00583 | LGG_00583 | Conserved protein |
| LGG_00584 | LGG_00584 | Conserved protein |
| LGG_00585 | LGG_00585 | Conserved protein |
| LGG_00586 | LGG_00586 | Putative protein without homology |
| LGG_00589 | LGG_00589 | Conserved protein |
| LGG_00593 | is32 | Transposase, IS5 family protein |
| LGG_00594 | LGG_00594 | Putative protein without homology |
| LGG_00628 | walR | Two-component response regulator |
| LGG_00629 | walK | Two component sensor transduction histidin |
| LGG_00630 | ycbN | ABC transporter, bacitracin transporter AT |
| LGG_00631 | LGG_00631 | ABC transporter, bacitracin transporter pe |
| LGG_00646 | LGG_00646 | Putative protein without homology |
| LGG_00647 | LGG_00647 | Putative protein without homology |
| LGG_00658 | LGG_00658 | Putative protein without homology |
| LGG_00659 | pts | PTS system, galactose-specific IIC compone |
| LGG_00660 | LGG_00660 | Putative protein without homology |
| LGG_00661 | LGG_00661 | Conserved protein |
| LGG_00662 | LGG_00662 | Beta-lactamase class C related penicillin |
| LGG_00663 | LGG_00663 | Conserved protein |
| LGG_00664 | lacC | Tagatose-6-phosphate kinase |
| LGG_00703 | LGG_00703 | Regulator of polyketide synthase expressio |
| LGG_00704 | LGG_00704 | Short-chain dehydrogenase/reductase SDR |
| LGG_00705 | adc | Acetoacetate decarboxylase |
| LGG_00813 | LGG_00813 | Conserved protein |
| LGG_00814 | LGG_00814 | Conserved protein |
| LGG_00815 | LGG_00815 | Conserved protein |
| LGG_00816 | LGG_00816 | Conserved protein |
| LGG_00817 | LGG_00817 | Conserved protein |
| LGG_03005 | 23S rRNA | 23S ribosomal RNA |
| LGG_00818 | LGG_00818 | Conserved protein |
| LGG_00819 | LGG_00819 | Lipoprotein |
| LGG_00820 | LGG_00820 | Conserved protein |
| LGG_00912 | is33 | Transposase, IS150/IS3 family protein |
| LGG_00913 | is34 | Transposase, IS3 family protein |
| LGG_00950 | LGG_00950 | Conserved protein |
| LGG_00965 | LGG_00965 | Conserved protein |
| LGG_00973 | LGG_00973 | Putative protein without homology |
| LGG_00974 | LGG_00974 | Conserved protein |
| LGG_01023 | LGG_01023 | Adenine specific DNA methylase Mod |
| LGG_01024 | is35 | Transposase |
| LGG_01025 | is36 | Transposase, IS4 family protein |
| LGG_01026 | LGG_01026 | Type III restriction-modification system m |
| LGG_01027 | res | Type III restriction-modification system r |
| LGG_01028 | LGG_01028 | Putative protein without homology |
| LGG_01029 | is37 | Transposase, IS5 family protein |
| LGG_01063 | LGG_01063 | Conserved protein |
| LGG_01064 | bglA | 6-phospho-beta-glucosidase (GH1) |
| LGG_01086 | int | Phage-related integrase |
| LGG_01087 | LGG_01087 | Putative protein without homology |
| LGG_01088 | LGG_01088 | Putative protein without homology |
| LGG_01089 | LGG_01089 | Conserved protein |
| LGG_01090 | LGG_01090 | Conserved protein |
| LGG_01091 | LGG_01091 | Putative protein without homology |
| LGG_01092 | LGG_01092 | Putative protein without homology |
| LGG_01093 | LGG_01093 | Putative protein without homology |
| LGG_01094 | LGG_01094 | Conserved protein |
| LGG_01095 | Cpg | Phage-related transcriptional regulator, C |
| LGG_01096 | LGG_01096 | Putative protein without homology |
| LGG_01097 | LGG_01097 | Putative protein without homology |
| LGG_01098 | LGG_01098 | Putative protein without homology |
| LGG_01099 | LGG_01099 | Conserved protein |
| LGG_01100 | LGG_01100 | Putative protein without homology |
| LGG_01101 | LGG_01101 | Conserved protein |
| LGG_01102 | LGG_01102 | Conserved protein |
| LGG_01103 | LGG_01103 | Putative protein without homology |
| LGG_01104 | LGG_01104 | RecT family protein |
| LGG_01105 | LGG_01105 | Phage-related protein |
| LGG_01106 | LGG_01106 | Phage-related replication protein |
| LGG_01107 | ssb3 | Single-stranded DNA-binding protein |
| LGG_01108 | LGG_01108 | Phage-related protein with HTH-domain |
| LGG_01109 | LGG_01109 | Phage-related protein DNA-binding protein |
| LGG_01110 | LGG_01110 | Phage-related protein |
| LGG_01111 | LGG_01111 | Phage-related protein |
| LGG_01112 | LGG_01112 | Phage-related holliday junction resolvase |
| LGG_01113 | LGG_01113 | Phage-related protein |
| LGG_01114 | LGG_01114 | Phage-related protein |
| LGG_01115 | LGG_01115 | Phage-related protein |
| LGG_01116 | LGG_01116 | Putative protein without homology |
| LGG_01117 | LGG_01117 | Phage-related protein |
| LGG_01118 | LGG_01118 | Putative protein without homology |
| LGG_01119 | LGG_01119 | Putative protein without homology |
| LGG_01120 | LGG_01120 | Putative protein without homology |
| LGG_01121 | LGG_01121 | Phage-related protein |
| LGG_01122 | LGG_01122 | Phage-related HNH endonuclease |
| LGG_01123 | LGG_01123 | Phage-related protein, ribonucleoside-diph |
| LGG_01124 | Rorf172 | Phage-related terminase, small subunit |
| LGG_01125 | Rorf447 | Phage-related terminase, large subunit |
| LGG_01126 | LGG_01126 | Phage-related portal protein |
| LGG_01127 | LGG_01127 | Phage-related Mu protein F like protein |
| LGG_01128 | LGG_01128 | Phage-related protein |
| LGG_01129 | gpG | Phage-related minor capsid protein (GpG pr |
| LGG_01130 | LGG_01130 | Phage-related protein |
| LGG_01131 | LGG_01131 | Phage-related protein |
| LGG_01132 | LGG_01132 | Phage-related head tail joining protein |
| LGG_01133 | LGG_01133 | Phage-related major structural protein |
| LGG_01134 | LGG_01134 | Phage-related major tail protein |
| LGG_01135 | LGG_01135 | Phage-related protein |
| LGG_01136 | LGG_01136 | Phage-related protein |
| LGG_01137 | LGG_01137 | Putative protein without homology |
| LGG_01138 | LGG_01138 | Phage-related tail component |
| LGG_01139 | LGG_01139 | Phage-related tail-host interaction protei |
| LGG_01140 | LGG_01140 | Phage-related protein |
| LGG_01141 | LGG_01141 | Phage-related protein |
| LGG_01142 | hol | Phage-related holin |
| LGG_01143 | lys | Phage-related lysin (GH25) |
| LGG_01150 | LGG_01150 | Putative protein without homology |
| LGG_01151 | LGG_01151 | Putative protein without homology |
| LGG_01152 | LGG_01152 | Putative protein without homology |
| LGG_01154 | LGG_01154 | Putative protein without homology |
| LGG_01186 | LGG_01186 | Putative protein without homology |
| LGG_01212 | LGG_01212 | Conserved protein |
| LGG_01236 | LGG_01236 | Conserved protein |
| LGG_01243 | LGG_01243 | Chromosome segregation ATPase |
| LGG_01244 | LGG_01244 | Putative protein without homology |
| LGG_01246 | LGG_01246 | Putative protein without homology |
| LGG_01247 | LGG_01247 | Putative protein without homology |
| LGG_01248 | is39 | Transposase, IS5 family protein |
| LGG_01250 | LGG_01250 | Conserved protein |
| LGG_01253 | LGG_01253 | Transcriptional regulator, xre family prot |
| LGG_01254 | LGG_01254 | DNA helicases |
| LGG_01316 | LGG_01316 | Putative protein without homology |
| LGG_01406 | LGG_01406 | Conserved protein |
| LGG_01515 | LGG_01515 | Integrase |
| LGG_01516 | LGG_01516 | Transcriptional regulator, xre family |
| LGG_01517 | LGG_01517 | Conserved protein |
| LGG_01518 | LGG_01518 | Putative protein without homology |
| LGG_01519 | LGG_01519 | Phage-related endolysin |
| LGG_01520 | LGG_01520 | Phage-related holin |
| LGG_01521 | LGG_01521 | Phage-related holin |
| LGG_01522 | LGG_01522 | Phage-related infection protein |
| LGG_01523 | LGG_01523 | Phage-related tail-host specificity protei |
| LGG_01524 | LGG_01524 | Phage-related tail component |
| LGG_01525 | LGG_01525 | Phage-related minor tail protein |
| LGG_01526 | LGG_01526 | Phage-related protein without homology |
| LGG_01527 | LGG_01527 | Phage-related tail component |
| LGG_01528 | LGG_01528 | Phage-related major tail protein |
| LGG_01529 | LGG_01529 | Phage-related tail component |
| LGG_01530 | LGG_01530 | Phage-related head-tail joining protein |
| LGG_01531 | LGG_01531 | Phage-related infection protein |
| LGG_01532 | LGG_01532 | Phage-related protein without homology |
| LGG_01533 | LGG_01533 | Phage-related prohead protease |
| LGG_01534 | LGG_01534 | Phage-related portal protein |
| LGG_01535 | LGG_01535 | Phage-related terminase large subunit |
| LGG_01536 | LGG_01536 | Phage-related terminase small subunit |
| LGG_01537 | tnpR | Phage-related Resolvase |
| LGG_01538 | LGG_01538 | Phage-related glycosyl transferase, group |
| LGG_01539 | is40 | Transposase, IS5 family protein |
| LGG_01540 | LGG_01540 | Phage-related HNH nuclease |
| LGG_01541 | ssb4 | Single-stranded DNA-binding protein |
| LGG_01542 | LGG_01542 | Putative protein without homology |
| LGG_01543 | LGG_01543 | Putative protein without homology |
| LGG_01544 | LGG_01544 | Putative protein without homology |
| LGG_01545 | rimL | Acetyltransferase, GNAT family protein |
| LGG_01546 | LGG_01546 | Conserved protein |
| LGG_01547 | LGG_01547 | ABC transporter, ATP-binding protein |
| LGG_01563 | LGG_01563 | ABC transporter, permease component |
| LGG_01564 | mppX | ABC transporter, ATP-binding protein |
| LGG_01579 | LGG_01579 | NADPH-quinone reductase (Modulator of drug |
| LGG_01580 | LGG_01580 | Transcriptional regulator, TetR family |
| LGG_01581 | is41 | Transposase, IS5 family protein |
| LGG_01582 | LGG_01582 | Oxidoreductase |
| LGG_01583 | LGG_01583 | Putative protein without homology |
| LGG_01584 | is42 | Transposase |
| LGG_01585 | is43 | Transposase, IS4 family protein |
| LGG_01586 | yohH | Glycosyl transferase, group 1 |
| LGG_01587 | yohJ | Glycosyl transferase, group 1 |
| LGG_01588 | LGG_01588 | Putative protein without homology |
| LGG_01589 | LGG_01589 | Cell surface protein |
| LGG_01590 | LGG_01590 | Conserved protein |
| LGG_01591 | LGG_01591 | Conserved membrane protein |
| LGG_01592 | LGG_01592 | Putative protein without homology |
| LGG_01593 | LGG_01593 | Conserved protein |
| LGG_01622 | is44 | Transposase, IS3/IS911 family protein |
| LGG_01623 | is45 | Transposase, IS150/IS3 family protein |
| LGG_01653 | oppC | ABC transporter, oligopeptide transporter |
| LGG_01707 | LGG_01707 | Conserved protein |
| LGG_01728 | LGG_01728 | Endopeptidase M23B |
| LGG_01729 | is46 | Transposase, IS605 family protein |
| LGG_01730 | LGG_01730 | Putative protein without homology |
| LGG_01748 | LGG_01748 | Transcriptional regulator, Rrf2 family |
| LGG_01749 | is47 | Transposase, IS5 family protein |
| LGG_01750 | LGG_01750 | Transporter, major facilitator superfamily |
| LGG_01751 | sir2 | NAD-dependent deacetylase, SIR2-like prote |
| LGG_01755 | LGG_01755 | Conserved protein |
| LGG_01843 | LGG_01843 | Putative protein without homology |
| LGG_01848 | LGG_01848 | Conserved protein |
| LGG_01866 | LGG_01866 | Transcriptional antiterminator |
| LGG_01881 | LGG_01881 | Conserved protein |
| LGG_01886 | LGG_01886 | Conserved protein |
| LGG_01887 | LGG_01887 | Lipoprotein |
| LGG_01888 | LGG_01888 | Conserved protein |
| LGG_01890 | LGG_01890 | Conserved protein |
| LGG_01891 | LGG_01891 | Conserved protein |
| LGG_01892 | LGG_01892 | Conserved protein |
| LGG_01905 | LGG_01905 | Fic family protein |
| LGG_01927 | LGG_01927 | Conserved transmembrane protein |
| LGG_01928 | LGG_01928 | Putative protein without homology |
| LGG_01936 | LGG_01936 | Alpha/beta hydrolase superfamily protein |
| LGG_01937 | gntR | Transcriptional regulator, GntR family |
| LGG_01938 | LGG_01938 | ABC transporter, ATPase component |
| LGG_01939 | LGG_01939 | ABC transporter, permease component |
| LGG_01940 | oppF | ABC transporter, oligopeptide transporter |
| LGG_01945 | oppA | ABC transporter, oligopeptide transporter |
| LGG_01950 | LGG_01950 | Type III restriction protein, res subunit |
| LGG_01951 | LGG_01951 | Aminoglycoside phosphotransferase |
| LGG_01952 | LGG_01952 | Zn-dependent endopeptidase, M10 family |
| LGG_01953 | LGG_01953 | Conserved protein |
| LGG_01954 | lciIC | Transcriptional regulator, xre family |
| LGG_01955 | LGG_01955 | Reverse transcriptase-like protein |
| LGG_01956 | is48 | Transposase, IS5 family protein |
| LGG_01957 | is49 | Transposase |
| LGG_01958 | LGG_01958 | Putative protein without homology |
| LGG_01959 | is50 | Transposase, IS4 family protein |
| LGG_01960 | LGG_01960 | Conserved protein |
| LGG_01961 | is51 | Transposase, IS66 family protein |
| LGG_01962 | LGG_01962 | Transposase |
| LGG_01963 | LGG_01963 | Conserved protein |
| LGG_01964 | LGG_01964 | Conserved protein |
| LGG_01965 | is52 | Transposase, IS5 family protein |
| LGG_01966 | LGG_01966 | Putative protein without homology |
| LGG_01967 | LGG_01967 | Conserved protein |
| LGG_01990 | LGG_01990 | Xylanase/chitin deacetylase |
| LGG_01991 | LGG_01991 | UDP-N-acetylglucosamine 2-epimerase |
| LGG_01992 | LGG_01992 | UDP-N-acetylglucosamine 2-epimerase |
| LGG_01993 | LGG_01993 | Conserved protein |
| LGG_01994 | LGG_01994 | Conserved protein |
| LGG_01995 | LGG_01995 | Conserved protein |
| LGG_01996 | rmlD | dTDP-4-dehydrorhamnose reductase |
| LGG_01997 | rmlB | dTDP-glucose 4,6-dehydratase |
| LGG_01998 | rmlC | dTDP-4-dehydrorhamnose 3,5-epimerase |
| LGG_01999 | rmlA | Glucose-1-phosphate thymidylyltransferase |
| LGG_02000 | LGG_02000 | Lyzozyme M1 (1,4-beta-N-acetylmuramidase) |
| LGG_02001 | LGG_02001 | Lyzozyme M1 (1,4-beta-N-acetylmuramidase) |
| LGG_02002 | LGG_02002 | Conserved protein |
| LGG_02003 | is53 | Transposase, IS5 family protein |
| LGG_02004 | eps3 | UDP-galactosephosphotransferase |
| LGG_02033 | is54 | Transposase, IS5 family protein |
| LGG_02038 | rmlB | dTDP-glucose 4,6-dehydratase |
| LGG_02039 | rmlC | dTDP-4-dehydrorhamnose 3,5-epimerase |
| LGG_02040 | rmlA1 | Glucose-1-phosphate thymidyl transferase ( |
| LGG_02041 | is55 | Transposase, IS5 family protein |
| LGG_02042 | rmlA2 | Glucose-1-phosphate thymidylyltransferase |
| LGG_02043 | welE | Undecaprenyl-phosphate beta-glucosephospho |
| LGG_02044 | welF | Glycosyl transferase, group 1 |
| LGG_02045 | welG | Glycosyl transferase,galactofuranosyltrans |
| LGG_02046 | welH | alpha-L-Rha alpha-1,3-L-rhamnosyltransfera |
| LGG_02047 | WelI | Glycosyl transferase, group 1 |
| LGG_02048 | welJ | Glycosyl transferase, alpha-1,3-galactosyl |
| LGG_02049 | wzx | Polysaccharide Transporter, PST family pro |
| LGG_02050 | glf | UDP-galactopyranose mutase |
| LGG_02051 | LGG_02051 | O antigen polymerase Wzy |
| LGG_02052 | wze | Tyrosine-protein kinase (capsular polysacc |
| LGG_02053 | wzd | Chain length regulator (capsular polysacch |
| LGG_02055 | LGG_02055 | Phage-related infection protein |
| LGG_02056 | LGG_02056 | Phage-related infection protein |
| LGG_02062 | oppF | ABC transporter, oligopeptide transporter |
| LGG_02063 | oppD | ABC transporter, oligopeptide transporter |
| LGG_02066 | oppA | ABC transporter, oligopeptide-binding prot |
| LGG_02087 | LGG_02087 | N-acetylmuramoyl-L-alanine amidase |
| LGG_02092 | LGG_02092 | Conserved protein |
| LGG_02093 | LGG_02093 | ATP-dependent Lon protease |
| LGG_02094 | LGG_02094 | Conserved protein (PglZ domain) |
| LGG_02095 | LGG_02095 | Adenine-specific methyltransferase, Type I |
| LGG_02096 | xerC | Phage-related integrase |
| LGG_02097 | LGG_02097 | Adenine-specific methyltransferase, Type I |
| LGG_02098 | LGG_02098 | Conserved protein |
| LGG_02099 | LGG_02099 | L-cystine import ATP-binding protein |
| LGG_02100 | LGG_02100 | Conserved protein |
| LGG_02160 | is56 | Transposase, IS4 family protein |
| LGG_02161 | is57 | Transposase |
| LGG_02165 | is58 | Transposase |
| LGG_02166 | is59 | Transposase, IS4 family protein |
| LGG_02171 | is60 | Transposase, IS5 family protein |
| LGG_02177 | LGG_02177 | Putative protein without homology |
| LGG_02178 | yosT | Phage-related DNA gyrase inhibitory protei |
| LGG_02199 | LGG_02199 | Putative protein without homology |
| LGG_02200 | LGG_02200 | Putative protein without homology |
| LGG_02201 | LGG_02201 | CRISPR-associated protein, SAG0897 family |
| LGG_02202 | cas2 | CRISPR-associated protein, Cas2 |
| LGG_02203 | cas1 | CRISPR-associated protein, Cas1 |
| LGG_02204 | csn1 | CRISPR-associated protein, Csn1 |
| LGG_02327 | LGG_02327 | Transcriptional regulator, xre family |
| LGG_02336 | LGG_02336 | ABC transporter, multidrug transporter ATP |
| LGG_02358 | LGG_02358 | Conserved protein |
| LGG_02359 | LGG_02359 | Conserved protein |
| LGG_02373 | LGG_02373 | Conserved protein |
| LGG_02376 | LGG_02376 | Transcriptional regulator, xre family |
| LGG_02380 | LGG_02380 | Prebacteriocin |
| LGG_02387 | hpk3 | Two component sensor transduction histidin |
| LGG_02427 | LGG_02427 | Conserved protein |
| LGG_02445 | is62 | Transposase, IS150/IS3 family protein |
| LGG_02446 | is63 | Transposase, IS3/IS911 family protein |
| LGG_02511 | LGG_02511 | Conserved protein |
| LGG_02512 | LGG_02512 | Conserved protein |
| LGG_02610 | LGG_02610 | Conserved protein |
| LGG_02611 | LGG_02611 | Conserved protein |
| LGG_02612 | LGG_02612 | Putative protein without homology |
| LGG_02613 | ABC-NBD | ABC transporter, ATP-binding protein |
| LGG_02614 | LGG_02614 | ABC transporter, ATP-binding protein |
| LGG_02651 | LGG_02651 | Transcriptional regulator, GntR family |
| LGG_02652 | LGG_02652 | Alpha-L-fucosidase (GH29) |
| LGG_02653 | pts | PTS system, IIAB component |
| LGG_02654 | levF | PTS system, IIC component |
| LGG_02655 | levG | PTS system, IID component |
| LGG_02656 | ubiD | 3-octaprenyl-4-hydroxybenzoate carboxy-lya |
| LGG_02657 | ubiX | 3-octaprenyl-4-hydroxybenzoate carboxy-lya |
| LGG_02662 | yniG | Transporter, major facilitator superfamily |
| LGG_02663 | LGG_02663 | Conserved protein |
| LGG_02664 | dgoD | Galactonate dehydratase |
| LGG_02665 | gatC | PTS system, galactitol-specific IIC compon |
| LGG_02666 | gatB | PTS system, galactitol-specific IIB compon |
| LGG_02667 | gatA | PTS system, galactitol-specific IIA compon |
| LGG_02668 | kdgA | 2-dehydro-3-deoxyphosphogluconate aldolase |
| LGG_02669 | LGG_02669 | Transcription antiterminator, BglG family |
| LGG_02670 | celC | PTS system, cellobiose-specific IIA compon |
| LGG_02671 | celA | PTS system, cellobiose-specific IIB compon |
| LGG_02672 | bglA | Beta-glucosidase (GH1) |
| LGG_02673 | ypbG | Sugar kinase and transkriptional regulator |
| LGG_02674 | ypdC | Conserved protein |
| LGG_02675 | LGG_02675 | Alpha-mannosidase (GH38) |
| LGG_02676 | is64 | Transposase, IS5 family protein |
| LGG_02677 | LGG_02677 | Alpha-mannosidase (GH38) |
| LGG_02678 | LGG_02678 | PTS system, cellobiose-specific IIC compon |
| LGG_02679 | gntR | Transcriptional regulator, GntR family |
| LGG_02680 | fcsR | Fucose operon repressor, DeoR family |
| LGG_02681 | LGG_02681 | Class II aldolase/adducin domain protein |
| LGG_02682 | fucU | L-fucose isomerase / RbsD or FucU transpor |
| LGG_02683 | ywtG | Transporter, major facilitator superfamily |
| LGG_02684 | fucK | Carbohydrate kinase, FGGY family |
| LGG_02685 | fucI | L-fucose isomerase |
| LGG_02686 | LGG_02686 | Putative protein without homology |
| LGG_02687 | rhaD | Rhamnulose-1-phosphate aldolase |
| LGG_02690 | rhaB | Rhamnulokinase |
| LGG_02694 | LGG_02694 | Conserved protein |
| LGG_02697 | is65 | Transposase, IS150/IS3 family protein |
| LGG_02698 | is66 | Transposase, IS3/IS911 family protein |
| LGG_02700 | LGG_02700 | Phage-related protein |
| LGG_02703 | LGG_02703 | Conserved protein |
| LGG_02742 | LGG_02742 | Conserved protein |
| LGG_02743 | xylB | Xylulokinase |
| LGG_02744 | LGG_02744 | Sorbitol dehydrogenase |
| LGG_02745 | esuD | fructose-bisphosphate aldolase |
| LGG_02746 | pts | PTS system, mannose/fructose/sorbose-speci |
| LGG_02747 | ahaB | PTS system, mannose/fructose/sorbose-speci |
| LGG_02748 | ahaA | PTS system, mannose/fructose/sorbose-speci |
| LGG_02749 | LGG_02749 | PTS system, mannose/fructose/sorbose-speci |
| LGG_02750 | is67 | Transposase, IS5 family protein |
| LGG_02751 | fbaA | Fructose-bisphosphate aldolase |
| LGG_02752 | LGG_02752 | Carbohydrate kinase, FGGY family |
| LGG_02753 | gatC | PTS system, galactitol-specific IIC compon |
| LGG_02754 | gatB | PTS system, galactitol-specific IIB compon |
| LGG_02755 | gatA | PTS system, galactitol-specific IIA compon |
| LGG_02756 | fba | Fructose-bisphosphate aldolase |
| LGG_02757 | farR | Transcriptional regulator, GntR family |
| LGG_02780 | LGG_02780 | Conserved protein |
| LGG_02870 | is68 | Transposase, IS3/IS911 family protein |
| LGG_02871 | is69 | Transposase, IS150/IS3 family protein |
| LGG_02874 | LGG_02874 | Conserved protein |
| LGG_02876 | LGG_02876 | Malate dehydrogenase |
| LGG_02877 | malP | Citrate carrier protein |
| LGG_02879 | dcuR | Two-component response regulator |
| LGG_02885 | xerC | Phage-related integrase |
| LGG_02886 | LGG_02886 | Transcriptional regulator |
| LGG_02887 | LGG_02887 | Putative protein without homology |
| LGG_02888 | LGG_02888 | Conserved protein |
| LGG_02889 | LGG_02889 | Conserved protein |
| LGG_02890 | LGG_02890 | Conserved protein |
| LGG_02891 | LGG_02891 | Conserved protein |
| LGG_02892 | LGG_02892 | Conserved protein |
| LGG_02893 | LGG_02893 | Phage-related protein, DNA replication |
| LGG_02894 | LGG_02894 | Phage-related virulence-associated protein |
| LGG_02895 | LGG_02895 | Phage-related protein |
| LGG_02896 | sb56 | Phage-related HNH endonuclease |
| LGG_02897 | terS | Phage-related terminase-small subunit |
| LGG_02898 | terL | Phage-related terminase large subunit |
| LGG_02899 | LGG_02899 | Phage-related conserved protein |
| LGG_02900 | LGG_02900 | Phage-related portal protein |
| LGG_02901 | LGG_02901 | Phage-related prohead protease |
| LGG_02902 | LGG_02902 | Phage-related head-to-tail joining |
| LGG_02903 | LGG_02903 | Putative protein without homology |
| LGG_02904 | LGG_02904 | Conserved extracellular protein |
| LGG_02905 | ytgB | Transglycosylase-associated protein |
| LGG_02930 | LGG_02930 | Conserved protein |
| LGG_02944 | tnp | Integrase |
