# Supplementary material for: Comparative Genomic and Functional Analysis of 100 Lactobacillus rhamnosus Strains and Their Comparison with Strain GG
Source: PLoS Genet. 2013 Aug 15;9(8):e1003683. doi: 10.1371/journal.pgen.1003683 (PMC3744422; doi:10.1371/journal.pgen.1003683)
Supplement: Table S4 — BLAST analysis of the spacers present in L. rhamnosus GG CRISPR locus. Each spacer was blasted using NCBI BlastN using the default parameters with the following modifications: word size 7, expected threshold 0.1, optimized for ‘somewhat similar’. (DOCX) [file pgen.1003683.s010.docx]

| **Spacer** | **Sequence** | **Length** | **ID number** | **Organism/bacteriophage/plasmid** | **Hit sequence** | **Host** |
| --- | --- | --- | --- | --- | --- | --- |
| 4 | TGAAATAATTGTTGACAC**G**TG**A**GAACACAG | 30 | gi\|77696193\|gb\|AY131267.2\| | Bacteriophage Lc-Nu | TGAAATAATTGTTGACAC**A**TG**G**GAACAC | *Lactobacillus rhamnosus Lc 1/3* |
| 4 | TGAAATAAT**T**GTTGACAC**G**TG**A**GAACACAG | 30 | gi\|166200914\|gb\|EU246945.1\| | Bacteriophage Lrm1 | TGAAATAAT**C**GTTGACAC**A**TG**G**GAACAC | *Lactobacillus rhamnosus M1* |
| 4 | TGAAATAAT**T**GTTGACACGTGAGAACACAG | 30 | gi\|22217797\|emb\|AJ251789.2\| | Bacteriophage A2 | TGAAATAAT**C**GTTGACACATGGGAACAC | *Lactobacillus casei ATCC 393* |
| 6 | TTAG**T**GGCA**G**AGAGGTAATGA**T**GATCACAC | 30 | gi\|77696193\|gb\|AY131267.2\| | Bacteriophage Lc-Nu | TTAG**C**GGCA**A**AGAGGTAATGA**G**GATCACAC | *Lactobacillus rhamnosus Lc 1/3* |
| 9 | GTCTAG**C**CC**G**AGAAGTTCCGCGGCTTTTTT | 30 | gi\|22217797\|emb\|AJ251789.2\| | Bacteriophage A2 | GTCTAG**T**CC**A**AGAAGTTCCGCGGCTTTT | *Lactobacillus casei ATCC 393* |
| 12 | CGCAGTCTTGCAAGCCGTAATGCTCGATCT | 30 | gi\|89953823\|gb\|DQ411856.1\| | Bacteriophage Lca1 | CGCAGTCTTGCAAGCCGTAATGCTCGAT | *Lactobacillus casei Lca1 - prophage* |
| 12 | CGCAGTCTTGCAA**G**CCGTAATGCTCGATCT | 30 | gi\|47607149\|gb\|AY605066.1\| | Bacteriophage φ AT3, | CGCAGTCTTGCAA**T**CCGTAATGCTCGATCT | *Lactobacillus casei* |
| 12 | CGCAGTCTTGCAAGCCGTAATGCTCGATCT | 30 | gi\|22217797\|emb\|AJ251789.2\| | Bacteriophage A2 | CGCAGTCTTGCAAGCCGTAATGCTCGAT | *Lactobacillus casei ATCC 393* |
| 18 | TAGTGATCA**T**ATTCATGCAACACAAACGGT | 30 | gi\|77696193\|gb\|AY131267.2\| | Bacteriophage Lc-Nu | TAGTGATCA**G**ATTCATGCAACACAAACGGT | *Lactobacillus rhamnosus Lc 1/3* |
| 18 | TAGTGATCA**T**ATTCATGCAACACAAACGGT | 30 | gi\|47607149\|gb\|AY605066.1\| | Bacteriophage φ AT3 | TAGTGATCA**G**ATTCATGCAACACAAACGGT | *Lactobacillus casei* |
| 21 | GGTGGCATCACAGAAGCAACCGAAACAATC | 30 | gi\|166200914\|gb\|EU246945.1\| | Bacteriophage Lrm1 | GGTGG**T**ATCACAGAAGCAACCGAAACAATC | *Lactobacillus rhamnosus M1* |
| 21 | GGTGG**C**ATCACAGAAGCAACCGAAACAATC | 30 | gi\|22217797\|emb\|AJ251789.2\| | Bacteriophage A2 | GGTGG**T**ATCACAGAAGCAACCGAAACAATC | *Lactobacillus casei ATCC 393* |
| 22 | AAGTGGTAAGGTCCCCAAAGATTCGATGAT | 30 | gi\|166200914\|gb\|EU246945.1\| | Bacteriophage Lrm1, | AAGTGGTAAGGTCCCCAAAGATTCGATGAT | *Lactobacillus rhamnosus M1* |
| 22 | AAGTGGTAAGGTCCCCAAAGA**T**TCGATGAT | 30 | gi\|687918\|gb\|S73384.1\| | Bacteriophage PL-1 | AAGTGGTAAGGTCCCCAAAGA**C**TCGATGAT | *Lactobacillus casei* |
| 22 | AAGTGGTAAGGTCCCCAAAGATTCGATGAT | 30 | gi\|22217797\|emb\|AJ251789.2\| | Bacteriophage A2 | AAGTGGTAAGGTCCCCAAAGATTCGAT | *Lactobacillus casei ATCC 393* |
| 24 | AAGCAATCCAGAAACTCGGGATCGTTTCTT | 30 | gi\|22217797\|emb\|AJ251789.2\| | Bacteriophage A2 | AAGCAATCCAGAAACTCGGGATCGTTTCTT | *Lactobacillus casei ATCC 393* |
| 14 | ACAGCCGTCTTGATCTTGTCAGCATCACCC | 30 | gi\|15722253\|emb\|AJ304453.1\| | Plasmid pSB102 | ACAGCCGTCTTGATCTTGTCAGCATCACCC* | *-* |

* Fragmented hit
